# Light‐Harvesting Photothermal Hotspots Enabled by NIR Scattering‐Absorption Coupling

**DOI:** 10.1002/advs.202523721

**Published:** 2026-03-09

**Authors:** Zebin Wu, Changming Bao, Pengyu Zhang, Tao Ding, Weiming Lin, Haodong Li, Wenjian Luo, Xizhi Deng, Hao Wang, Guanle Li, Hongyue Hu, Ye Wang, Dawei Jiang, Yu Zhang, Sisi Jia, Nan Zhang, Min Ke, Le Liang

**Affiliations:** ^1^ The Institute for Advanced Studies (IAS) Department of Ophthalmology Zhongnan Hospital of Wuhan University State Key Laboratory of Metabolism and Regulation in Complex Organisms College of Life Sciences Wuhan University Wuhan China; ^2^ Department of Ophthalmology Zhongnan Hospital of Wuhan University Wuhan China; ^3^ Department of Nuclear Medicine Union Hospital Tongji Medical College Huazhong University of Science and Technology Wuhan China; ^4^ The Interdisciplinary Research Center Shanghai Synchrotron Radiation Facility Zhangjiang Laboratory Shanghai Advanced Research Institute Chinese Academy of Sciences Shanghai China; ^5^ Zhangjiang Laboratory Shanghai China

**Keywords:** endocytosis‐driven hotspots, gold nanostars, low‐power photothermal ablation, nanodiamonds, photothermal conversion, scattering–absorption coupling

## Abstract

Efficient near‐infrared (NIR) photothermal conversion is constrained by photon loss from scattering and attenuation in complex media. We present a scattering–absorption coupling strategy that pairs broadband photon scattering with plasmonic absorption to deliver supra‐additive heating. Nanodiamonds (ND) multiply scatter and trap NIR photons, elevating local photon density, while neighboring gold nanostars (AuNS) convert the enriched flux into heat with high efficiency. A multi‐scale framework links mechanism to outcome: finite‐difference time‐domain calculations quantify AuNS absorbed‐power gains in the proximity of ND, and Monte Carlo photon transport yields a depth‐dependent photon‐density enhancement that together predicts the measured temperature rises. In agarose phantoms and bacterial plates, ND increases NIR absorption and, when combined with AuNS, produces greater‐than‐additive heating. In cells, endogenous endocytosis brings ND and AuNS into lysosomal microenvironments that act as confined “hotspots” enabling low‐power ablation; the power density required for 50% cell kill (LD_50_) is reduced by a factor of 4.2 relative to AuNS alone. In in vivo experiments, AuNS and ND also exhibited a more potent photothermal therapeutic effect on mouse tumors. This principle provides a design rule for coupling scatterers and absorbers to overcome photon scarcity, offering a biocompatible route to efficient photothermal action with potential across oncology, anti‐infective therapy, and other light‐managed applications.

## Graphical abstract

By coupling the broadband scattering of nanodiamonds with the plasmonic absorption of gold nanostars, this work proposes a novel strategy for the active modulation of photon transport. This strategy creates localized photothermal hotspots in turbid media and achieves a supra‐additive heating efficiency, thereby providing a universal platform for low‐power and high‐efficiency photothermal therapy and biological modulation.

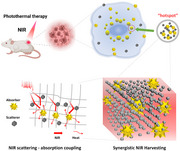

## 1 Introduction

Efficient conversion of near‐infrared (NIR) light into heat remains a central challenge for light‐managed processes that demand precise, localized heat in optically complex media. Including on‐chip thermal actuation, phase‐change control, microreactor catalysis, soft‐matter processing, controlled release, biosensing, and biointerface modulation, in addition to therapeutic and antimicrobial uses [1, 2, 3, 4, 5, 6, 7, 8, 9, 10]. In such turbid environments, multiple scattering and attenuation rapidly deplete the usable photon budget at the target site, so that robust heating typically requires elevated laser powers or high nanoparticle doses. These constraints motivate a shift in emphasis from “adding more absorption” to managing the local photon supply: prolonging photon residence time and recycling incident NIR light where heat must be generated, while preserving biocompatibility and architectural simplicity [11, 12, 13, 14, 15].

Existing remedies generally follow three axes—raising absorber loading [16], engineering larger effective absorption cross‐sections (e.g., plasmonic reshaping) [17, 18], or increasing laser power [19]—each with trade‐offs in safety, fabrication complexity, or thermal management [20]. Gold nanostars (AuNS) are compelling absorbers because their pronounced NIR‐localized surface plasmon resonance (LSPR) enables strong light–matter coupling and rapid heat generation [21, 22, 23, 24, 25, 26], yet AuNS alone often require high local concentration [27] or surface engineering to achieve sufficient on‐target heating. In parallel, nanodiamonds (ND), widely used for thermal transport [28, 29, 30, 31] and imaging [32, 33, 34, 35, 36], exhibit intrinsic broadband scattering that can trap photons by extending their path length, thereby elevating local photon density even at low loadings [37]. What is missing is a quantitative coupling framework that links scatterer‐mediated photon management to absorber heating—and ultimately to functional outcomes—under realistic constraints of proximity, dose, and medium turbidity. Such a framework would provide design rules, specifying how scatterer number, absorber–scatterer spacing, and optical coefficients jointly determine thermal response at a given dose, rather than case‐specific recipes.

Here, we establish and validate that framework and instantiate it with nanodiamonds (broadband scatterers) and gold nanostars (efficient absorbers). Finite‐difference time‐domain (FDTD) simulations quantify how sub‐50‐nm proximity to ND redistributes the near field and increases the absorbed power of adjacent AuNS; mesoscopic Monte‐Carlo photon transport shows that ND scattering elevates the depth‐dependent photon density in turbid matrices, supplying the photons that AuNS harvest under identical doses. Together with an analytical heat‐balance description, these elements form a predictive model that turns qualitative “two‐component synergy” into a quantitative, designable principle. We then translate the mechanism into practice: Co‐embedding AuNS and ND yields greater‐than‐additive temperature rises in agarose phantoms and enhanced low‐power sterilization of Staphylococcus aureus; in cells, endogenous endocytosis brings both components into lysosomal microenvironments that function as confined optical “hotspots,” confirmed by in situ single‐particle spectroscopy and quantitative imaging (Pearson R ≈ 0.70). Functionally, the power density required for 50% tumor‐cell ablation drops from 143 to 34 mW cm^−^^2^ (≈4.2‐fold reduction) relative to AuNS alone, with negligible dark toxicity. In vivo, AuNS and ND can also co‐localize within mouse tumors via co‐injection, thereby achieving a more potent photothermal therapeutic effect against tumors. By managing photons rather than merely adding absorbers or power, the coupling strategy provides a generalizable route to efficient low‐dose photothermal action across diverse light‐managed technologies—from microreactors and phase‐change systems to biosensing and biointerfaces—while retaining simplicity and biocompatibility.

## 2 Results and Discussion

### 2.1 Framework for Synergistic Near‐Infrared (NIR) Light Harvesting

**FIGURE 1:**
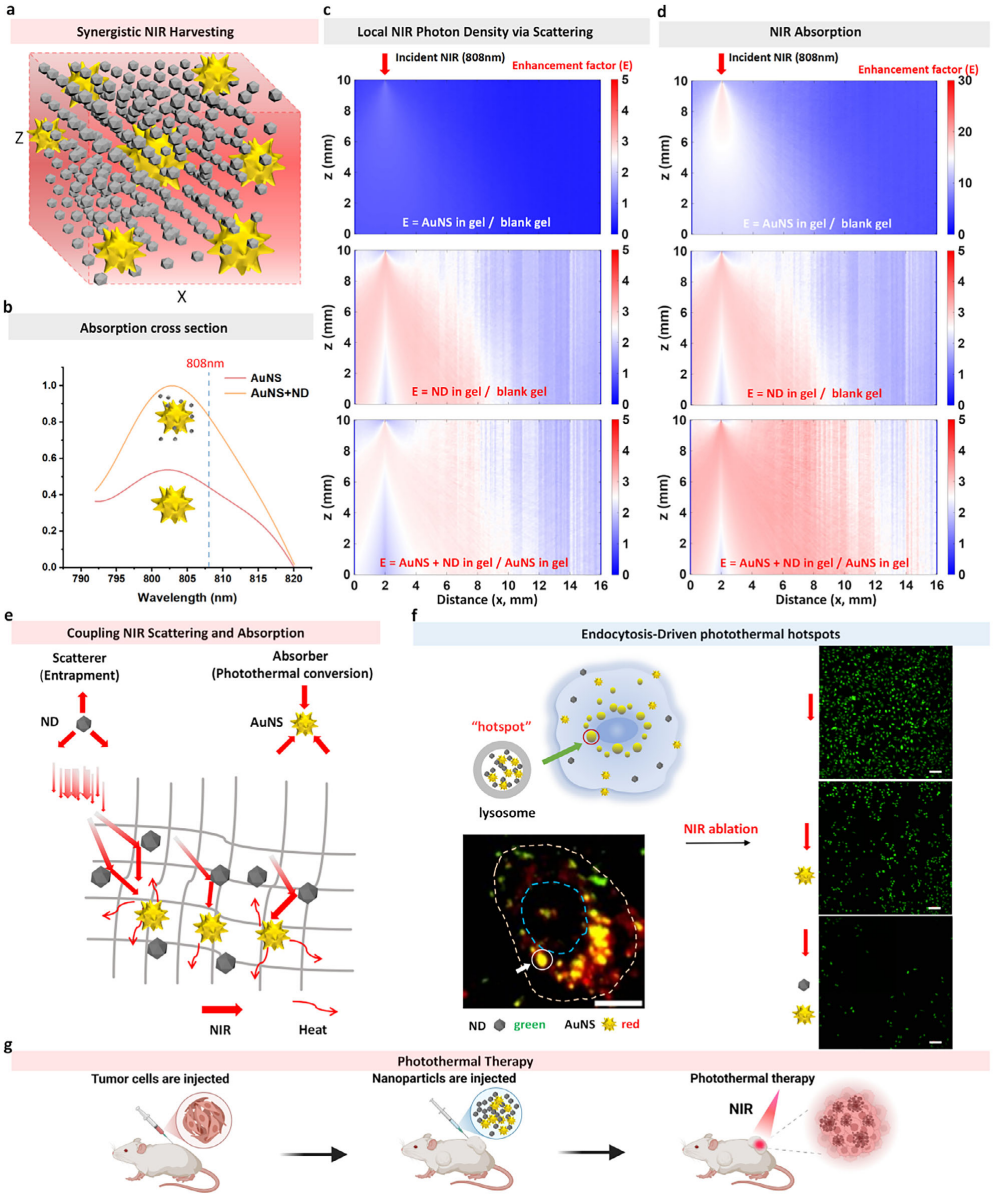
Computational‐experimental framework for synergistic NIR light harvesting by AuNS‐ND hybrids. (a) 3D modeling diagram of the coexistence of AuNS and ND in the media. (b) Absorption cross‐sections of AuNS in two systems, namely AuNS and AuNS+ND, simulated by FDTD. The blue dashed line represents the absorption cross‐section at 808 nm. (c) Monte‐Carlo photon‐transport maps of the photon‐density enhancement factor. Distribution map of local NIR photon density enhancement coefficients under the comparison of different systems, obtained by dividing the photon density of the former by that of the latter. The depth of color represents the enhancement coefficient. The z‐axis is depth from the illuminated face (top→bottom), and the x‐axis is lateral position. Incident 808 nm light enters from the top. (d) Corresponding maps of absorption enhancement. NIR absorption enhancement coefficients under the comparison of different systems, obtained by dividing the near‐infrared absorption of the former by that of the latter. Axes, illumination, and color scale as in (c,e). Conceptual illustration of the complementary optical roles played by nanodiamonds (ND, grey spheres) and gold nanostars (AuNS, yellow stars). ND multiply scatter and trap incident NIR photons (red arrows), while AuNS strongly absorb the trapped light and convert it to heat, yielding supra‐additive photothermal output. (f) Schematic diagram of the endocytic pathway that drives AuNS and ND into late endosomes/lysosomes, where they aggregate to form local photothermal “hotspots”. Fluorescence images demonstrate that the photothermal ablation effect is enhanced after intracellular aggregation of AuNS and ND. Bottom left: Confocal superimposed image of representative cells, showing the co‐localization of ND (green channel) and AuNS (red channel) in lysosomal compartments (white arrows), scale bar: 10 µm. Right images from top to bottom: untreated control group, AuNS‐only treated group, AuNS+ND treated group after near‐infrared irradiation. Viable cells emit green fluorescence (calcein‐AM); the disappearance of green signals indicates cell death, scale bar: 100 µm. (g) Schematic diagram of photothermal therapy for a mouse tumor.

To quantify how scattering and absorption couple to manage NIR photons in turbid media, we combined finite‐difference time‐domain (FDTD) electrodynamics with Monte Carlo (MC) photon transport on a 3D gel phantom containing both AuNS and ND (Figure 1a) (All detailed simulation methods are described in the supporting information, and the parameters used are listed in Table S1). FDTD first showed that placing ND within sub‐50‐nm proximity of an AuNS increases the effective absorption cross‐section/absorbed power near 808 nm (Figure 1b). The effect is strongly distance‐dependent: ND in the near field of the AuNS spike region produces a pronounced increase, whereas remote ND has a negligible impact (Figure S1). This indicates that ND‐mediated near‐field redistribution and local photon recycling can make more optical energy available to the plasmonic absorber under identical incident dose.

We next examined transport‐level consequences in millimeter‐scale media. MC simulations on cylindrical gels revealed that ND substantially reshape photon trajectories by repeated scattering, prolonging their path length and residence time; by contrast, blank gels or gels containing only AuNS show far fewer, more ballistic paths (Figure S2). The photon‐density enhancement map in Figure 1c quantifies this effect. Here we define the enhancement factor E(x,z) as the ratio:

$$E=\Phi_{\mathrm{case}\;(x,\;z)}\;/\;\Phi_{\mathrm{reference}\;(x,\;z)}$$

where Φ denotes the local photon density (fluence rate; i.e., time‐averaged photon energy flux per unit volume within the medium, reported in normalized arbitrary units with respect to the incident flux). Relative to blank gel, ND in gel yields multi‐fold increases in Φ throughout the illuminated volume, with the highest gains (≥ 4 ×) near the entry side. AuNS in gel alone shows E ≤ 1 little enhancement vs. gel because absorption depletes photons rather than recycles them. Importantly, the AuNS+ND in gel / AuNS in gel map remains a significant enhancement (E > 1over a broad region), demonstrating that ND elevates the photon supply available to adjacent AuNS even when absorption is present. These findings confirm that ND‐driven multiple scattering traps photons, extending their residence time and creating a high‐flux microenvironment.

Consistently, the absorption‐enhancement maps in Figure 1d (defined as the ratio of local absorbed power/energy density, equivalently μ_*a*_× Φ (μ_*a*_represents the absorption coefficient), between the compared cases) show higher local absorption for ND in gel than gel and for AuNS+ND in gel than AuNS in gel, corroborating the FDTD‐predicted increase in absorption cross‐section of AuNS when ND are proximal. Together, these simulations establish a two‐step mechanism: ND‐driven multiple scattering increases local photon density; proximal AuNS harvests that enriched flux with high efficiency.

To ensure the reliability and robustness of the model, we further conducted a systematic sensitivity analysis on a series of key optical parameters that influence the photothermal response, including the background absorption coefficient µ_a,bkg_​, background scattering coefficient µs_,bkg_​, anisotropy factor g, refractive index n, AuNS absorption coefficient µ_a,AuNS_​, and ND scattering coefficient µs_,ND_​. This analysis was designed to identify the parameters exerting the most significant influence on the outputs and verify the stability of the model's predicted results under fluctuations of different parameters. The results (Figure S3) show that the global absorption rate of the system is highly sensitive to µ_a,AuNS_​, moderately sensitive to g and n, and exhibits low sensitivity to the background parameters µ_a,bkg​_ and µs_,bkg_​. This trend confirms that the absorption behavior is dominated primarily by the additional absorption induced by nanoparticles, rather than the optical properties of the background medium. Notably, the ND scattering coefficient µs_,ND_​ has no significant effect on the global absorption rate. We hypothesize that this is mainly because ND alter the spatial distribution of photons primarily through scattering, rather than participating in absorption directly. To verify this mechanism, we further performed a sensitivity analysis of the spatial light distribution targeting µs_,ND_​. The results clearly demonstrate (Figure S4) that the local light distribution exhibits significant sensitivity to variations in µs_,ND_​, indicating that although ND do not absorb photons directly, they can effectively modulate the local optical field through scattering, thereby enhancing the light‐harvesting efficiency of adjacent AuNS and ultimately synergistically boosting the overall photothermal performance of the system.

Guided by the simulation results, we integrate AuNS and ND into a unified, self‐driven nanosystem (Figure 1e). AuNS serve as absorbers/primary photothermal transducers, whereas ND act as scatterers/photon recyclers that trap NIR light; collectively, they raise local photon density and yield supra‐additive heating. In cells, endogenous endocytosis brings both components into the same lysosomal compartments, generating confined optical “hotspots” where enhanced photon availability and strong AuNS absorption combine to boost photothermal conversion (Figure 1f). Confocal overlays show co‐localization of ND (green) and AuNS (red), and viability imaging confirms stronger NIR‐induced ablation than either component alone. This self‐assembly route eliminates the need for targeting ligands, maintains biocompatibility, and provides the nanoscale proximity required for effective scattering–absorption coupling. In vivo, AuNS and ND can also exert a stronger therapeutic effect for mouse tumor photothermal therapy through their synergistic effect (Figure 1g).

### 2.2 Nanoparticle‐Enabled NIR Harvesting

**FIGURE 2:**
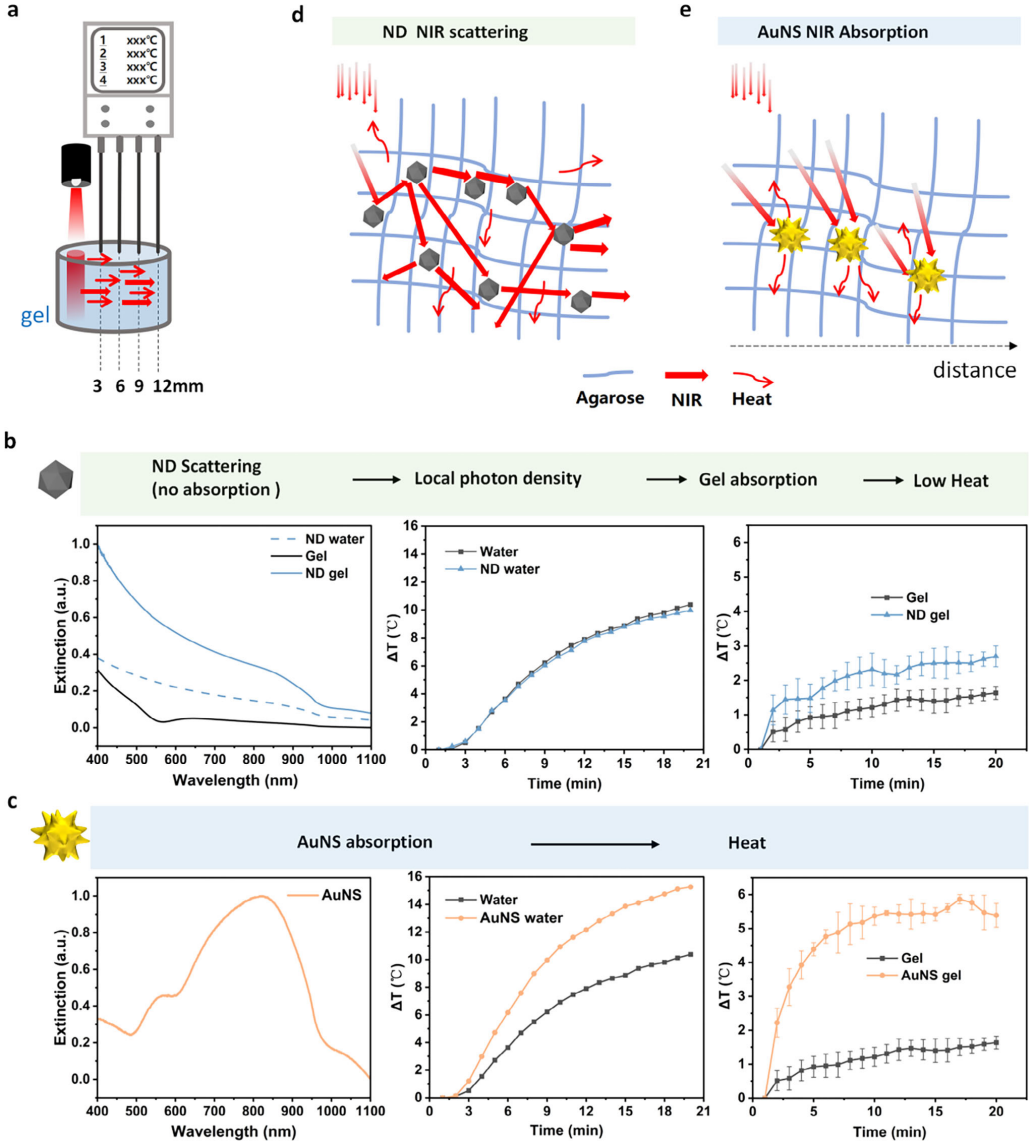
Nanoparticle‐enabled NIR harvesting. AuNS: 3.6 pM, ND: 3.2 × 10^−^^4^, 3.2 × 10^−^^3^, and 9.6 × 10^−^^3^v% (11.2, 112, and 336 µg/ml). Incubation time: 30 min. (a) Experimental set‐up: an 808 nm laser irradiates (power: 500 mW, height: 5 cm) one end of a nanoparticle‐laden agarose cylinder (2 wt.%), while four 1 mm‐tip thermocouples—spaced 3 mm apart, the nearest positioned 3 mm from the beam—record temperature profiles over 20 min. (b) ND scattering, ND: 3.2 × 10^−^3 v% (112 µg/ml). Left: UV–vis spectra of agarose gel (black), ND suspensions in water (dashed blue), and ND‐laden gels (solid blue). Middle: Temperature rise (ΔT) for water (black) vs. ND suspensions (blue) (power: 2 W, height: 10 cm). Right: ΔT for neat gel (black) vs. ND‐laden gels (blue) at 3 mm from the beam (n = 3). (c) AuNS absorption, AuNS: 3.6 pM. Left: extinction spectrum of AuNS showing a pronounced LSPR peak centred at ∼ 800 nm (orange). Middle: ΔT for AuNS suspension (orange) vs. water (black) (power: 2 W, height: 10 cm). Right: ΔT for AuNS‐laden (orange) gel vs. neat gel (black) (n = 3). (d) Schematic illustrating how densely distributed ND multiply scatter and trap incident NIR photons, elevating local photon density and thereby enhancing gel heating. (e) Schematic illustrating AuNS‐dominated absorption‐to‐heat conversion, which produces rapid, high‐amplitude temperature rises. Error bars represent the standard deviation of three independent samples. Data are mean ± SD.

Agarose gel (2 wt. %)—chosen for its optical clarity, thermal stability, and mechanical strength [38, 39]—served as a solid phantom to quantify NIR‐to‐heat conversion by individual nanoparticle formulations. Unmodified nanodiamonds (ND, ∼10 nm) and gold nanostars (AuNS) were dispersed in boiling agarose, cast into well plates, and demoulded to yield uniformly loaded cylinders (Figure S6). TEM confirmed the size homogeneity of ND and the branched morphology of AuNS (Figures S7 and S8). During measurement, an 808 nm laser irradiated one end of each gel, while four 1 mm‐tip thermocouples—spaced 3 mm apart, the nearest 3 mm from the beam—recorded temperature for 20 min (Figures 2a; S9).

Control experiments highlighted the distinct optical roles of the two nanoparticles. UV–vis spectrophotometry revealed that the nanodiamond (ND) suspension exhibits essentially featureless extinction in the near‐infrared region, with no discernible characteristic NIR extinction peak. Moreover, compared with pure water, 808‐nm NIR laser irradiation induced a negligible temperature rise in the ND dispersion, indicating intrinsically weak absorption of 808‐nm light by ND (Figure 2b, left and middle). When embedded in agarose, however, the gel's absorbance at 808 nm rose sharply (Figure 2b, left). Accordingly, compared with the blank hydrogel, the nanodiamond‐loaded hydrogel exhibited a substantially greater temperature rise under 808‐nm NIR laser irradiation (Figure 2b, right). This behavior was reproduced at every thermocouple position (Figure S10), with radial ΔT decaying as expected for conductive heat loss (Figure S11). Across three ND loadings (3.2 × 10^−^^4^, 3.2 × 10^−^^3^, 9.6 × 10^−^^3^ v %, corresponding to mass concentrations of 11.2, 112, and 336 µg/ml), ND‐laden gels consistently showed higher absorbance at 808 nm than matching ΔT rises (Figures 2b, left; S12). Because ND in suspension does not heat appreciably, intrinsic absorption alone cannot account for the enhanced gel temperatures. Instead, densely distributed ND multiply scatter incoming NIR photons (Figure 2d), lengthening their optical path, elevating local photon density, and allowing the agarose matrix to absorb and convert more energy. Increasing ND content intensifies this light‐entrapment effect, yielding progressively larger temperature rises [37].

To more clearly elucidate the optical role of ND in our system, we performed UV–vis measurements using an integrating‐sphere configuration. Conventional cuvette‐based normal‐incidence transmission measurements report only the optical extinction (apparent absorbance). In highly turbid nanodiamond dispersions, however, strong scattering deflects photons away from the forward detection path, which can be readily misinterpreted as absorption. In contrast, an integrating sphere collects diffusely scattered light over a wide range of angles in both reflectance and transmittance geometries, thereby minimizing scattering‐related artifacts and enabling a more accurate quantification of the intrinsic absorption contribution of nanodiamonds. Using this approach, we measured the wavelength‐dependent diffuse reflectance of nanodiamond powders (Figure S13). The nanodiamonds exhibited a consistently high diffuse reflectance across the visible–near‐infrared range (400–1100 nm), increasing from ∼88% at 400 nm to ∼98% at 1100 nm. Notably, around 808 nm (the laser wavelength used throughout this study), the reflectance remained above 93%, indicating that ND exhibits extremely strong scattering behavior and the intrinsic NIR absorption of nanodiamonds is negligible. Collectively, these results demonstrate that nanodiamonds primarily act as scatterers that redistribute incident photons (increase the effective optical path length and local photon density), rather than directly contributing to photothermal conversion via absorption. Meanwhile, simulation calculations also show that the absorption cross‐section of ND (C_abs​_) is approximately zero, which indicates that the optical contribution of nanodiamonds is dominated primarily by scattering.

AuNS functioned as strong NIR absorbers, displaying a pronounced localized surface‐plasmon resonance (LSPR) peak at ∼ 800 nm (Figure 2c, left). Under identical 808 nm irradiation, both AuNS suspensions and AuNS‐laden gels heated rapidly, reaching plateaus far higher than those of ND‐laden gels (Figure 2c, middle and right). As illustrated in Figure 2e, this rapid temperature rise originates from AuNS's direct absorption‐to‐heat conversion, underscoring their role as the primary photothermal transducers in the gel matrix.

In summary, ND act as photon scatterers that intensify local photon density, whereas AuNS serve as efficient absorbers that convert those photons into heat. These complementary functions establish a mechanistic foundation for the synergistic NIR harvesting explored in the following sections.

### 2.3 Synergistic Energy Harvesting by Co‐Embedded Nanodiamond (ND) and Gold Nanostar (AuNS)

**FIGURE 3:**
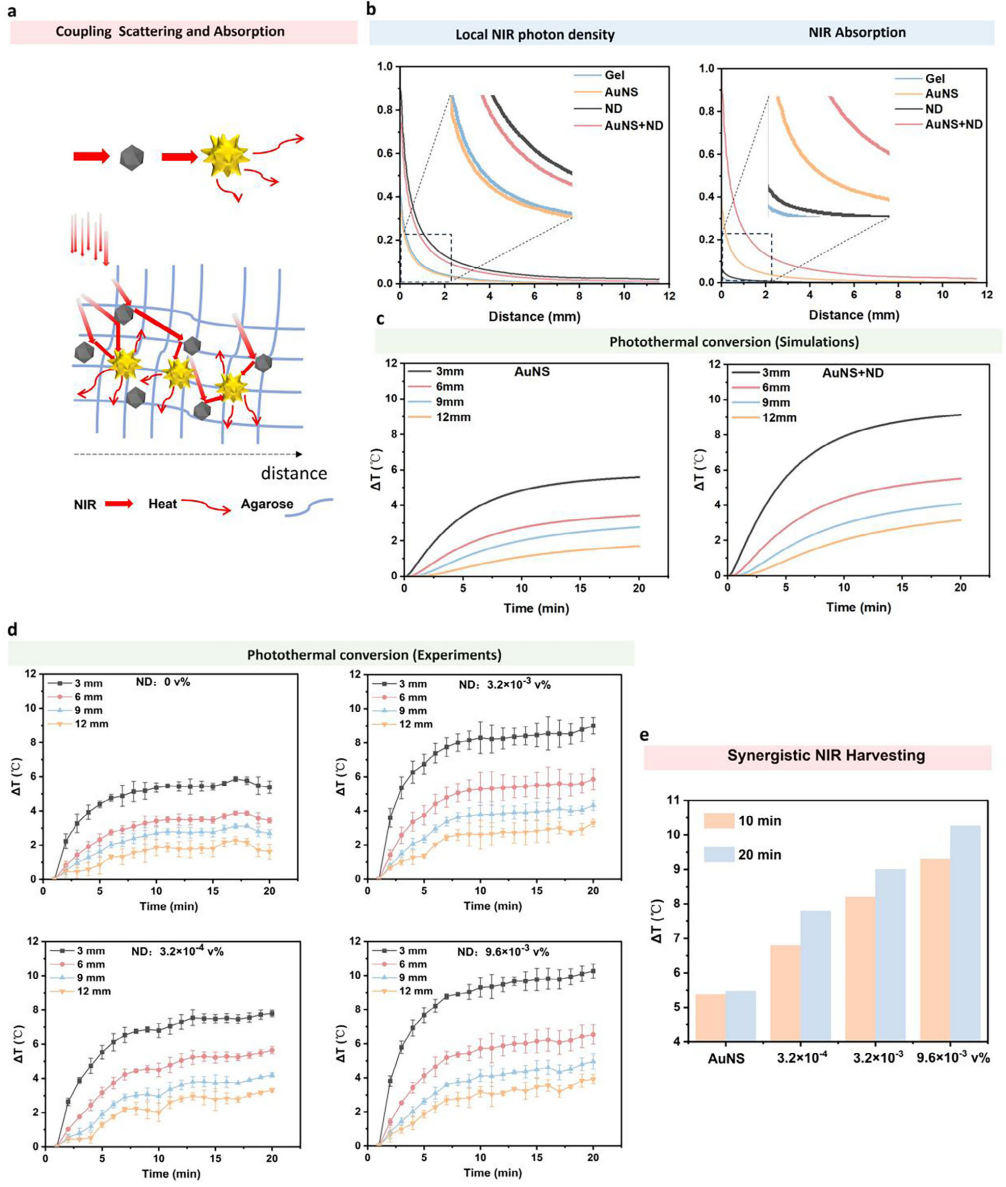
Synergistic NIR harvesting in agarose gels co‐loaded with AuNS and ND. AuNS: 3.6 pM, ND: 3.2 × 10^−^^4^, 3.2 × 10^−^^3^, and 9.6 × 10^−^^3^ v %. Power: 500 mW, Time: 20 min, Height: 5 cm. Incubation time: 30 min. (a) Schematic of the cooperative mechanism: ND (grey) scatter and trap NIR photons, increasing the photon flux incident on AuNS (yellow), which absorb the light and convert it into heat. (b) Statistical results of local near‐infrared photon density and near‐infrared absorption in different systems. (c) Thermal analysis temperature simulation curves of AuNS and AuNS+ND gel models. (d) Temperature rise (ΔT) as a function of time at four probe distances (3, 6, 9, 12 mm) for gels containing a constant AuNS concentration (3.6 pM) and four ND volume fractions (0, 3.2 × 10^−^^4^, 3.2 × 10^−^^3^, 9.6 × 10^−^^3^ v %). Data are mean ± SD (n = 3). (e) Bar chart summarising ΔT at the 3 mm position after 10 min (orange) and 20 min (blue) of irradiation, highlighting the ND‐concentration‐dependent enhancement of the photothermal effect.

Having shown that ND raises the local NIR photon supply and that AuNS acts as an efficient photothermal transducer, we co‐embedded ND and AuNS in agarose to test whether ND‐mediated scattering further elevates the photothermal response (Figure 3a). Before experimentation, we quantified the distance dependence of the NIR photon density and NIR absorption at different positions from the light source along the position indicated by the dotted line (Figure S5). The profiles (Figure 3b) show that ND in gel exhibits the highest local photon density Φ across the illuminated volume, consistent with multiple scattering and photon trapping. Because AuNS absorb photons, Φ in AuNS+ND is lower than in ND but higher than in AuNS and blank gel, indicating that ND still raises the photon supply available to nearby AuNS under the same incident dose. The corresponding absorption profiles mirror this trend: relative to their references, both ND vs gel and AuNS+ND vs AuNS display clear absorption enhancement, in agreement with the near‐field FDTD prediction that proximal ND increases the absorbed power of adjacent AuNS.

Cylindrical gels were prepared with a fixed AuNS concentration (3.6 pM) while the ND volume fraction was stepped from 0 to 9.6 × 10^−^^3^ v %. Upon 808 nm irradiation, the temperature rise (ΔT) was tracked at 3, 6, 9, and 12 mm from the laser beam for 20 min (Figure 3d). Across all four probe distances, the presence of ND uniformly shifted the heating curves upward. At the most responsive position (3 mm), the AuNS‐only gel reached ΔT ∼5.5°C after 20 min, whereas gels containing 3.2 × 10^−^^4^, 3.2 × 10^−^^3^, and 9.6 × 10^−^^3^ v % ND attained ∼ 8, 9, and 10.5°C, respectively—corresponding to incremental gains of ∼ 40%, 60%, and 90%. A similar concentration‐dependent enhancement persisted at 6, 9, and 12 mm, although absolute values decreased with distance because of radial heat dissipation (Figure S14).

For a concise comparison, ΔT values at 3 mm were extracted at 10 min (rapid‐heating phase) and 20 min (quasi‐steady state). As summarized in Figure 3e, introducing the smallest ND fraction already lifted ΔT from ∼5°C to ∼7°C at 10 min; at the highest ND loading, the rise doubled to ∼9°C. After 20 min, the same series increased ΔT from ∼5.5°C (AuNS only) to ∼10.5°C—a clear supra‐additive effect that cannot be explained by simply summing the individual contributions of ND and AuNS. To quantify the data, we defined a standardized evaluation index, the synergy coefficient (S_ΔT_)

$$\;S_{\Delta T}=\frac{\Delta T_{\mathrm{AuNS}+\mathrm{ND}}}{\Delta T_{\mathrm{AuNS}}+\Delta T_{\mathrm{ND}}}$$

A synergistic effect between NDs and AuNSs is considered to exist when S_ΔT_ >1. The ΔT values measured at a depth of 3 mm for 20 min with an ND concentration of 3.2 × 10^−^^3^ v% are presented as typical data. Specifically, the ΔT_ND_ of the ND gel at 3 mm was 2.6°C, the ΔT_AuNS_ of the AuNS gel was 5.38°C, and the ΔT_AuNS+ND_ of the AuNS+ND gel reached 9.04°C, yielding an experimental S_ΔT_ >1. Furthermore, we calculated the S_ΔT_ values at different ND concentrations for both 10‐min and 20‐min irradiation durations, and all of these values were greater than 1. Thus, a synergistic effect can be confirmed to exist between the ND and AuNS components.

For further validation, we used a spectrophotometer to measure the Extinction changes of AuNS‐laden gels after the incorporation of ND, so as to verify whether the light‐harvesting capacity was enhanced. The results (Figure S15) showed that the LSPR peak of AuNS‐laden gels at ∼808 nm increased significantly after adding ND, and the peak intensity further elevated with the increase of ND concentration. Therefore, it can be illustrated that the incorporation of ND into AuNS‐laden gels significantly enhances the NIR light absorption capacity of AuNS, which is attributed to the NIR light scattering effect of ND. It can be concluded that the NIR light‐harvesting capacity of the hybrid system is improved.

To cross‐validate, we performed thermal analysis temperature simulations on the AuNS and AuNS+ND models (Figure 3c). The results showed that the temperature curves of the AuNS group and the 0 v% group, as well as those of the AuNS+ND group and the 3.2 × 10^−^^3^ v% group, are very similar, with almost the same ΔT at 10 and 20 min. This agreement between transport modeling, near‐field electrodynamics, and bulk thermometry supports a coherent mechanism: ND multiply scatter and trap incident photons, increasing local photon density; proximal AuNS harvest this enriched flux and convert it to heat with high efficiency. As ND concentration increases, more photons are retained, reinforcing a positive interplay between scattering (ND) and absorption (AuNS) that manifests as faster heating rates and higher steady‐state temperatures. Thus, coupling these two optical pathways provides a simple, ligand‐free route to amplify photothermal efficiency without complex nanostructuring.

### 2.4 Synergistic Photothermal Antibacterial Activity

Post‐operative wound infections, especially those caused by *Staphylococcus aureus* (*S. aureus*), are a principal source of complications and delayed healing [40, 41, 42]. Photothermal therapy (PTT), recognized for its efficacy and minimal side effects, has garnered extensive research attention as a sterilization method in antibacterial treatments [43, 44]. Because co‐embedded AuNS and ND harvest NIR energy more efficiently than either component alone, we hypothesized that this coupling would translate into superior bactericidal efficacy under NIR irradiation.

**FIGURE 4:**
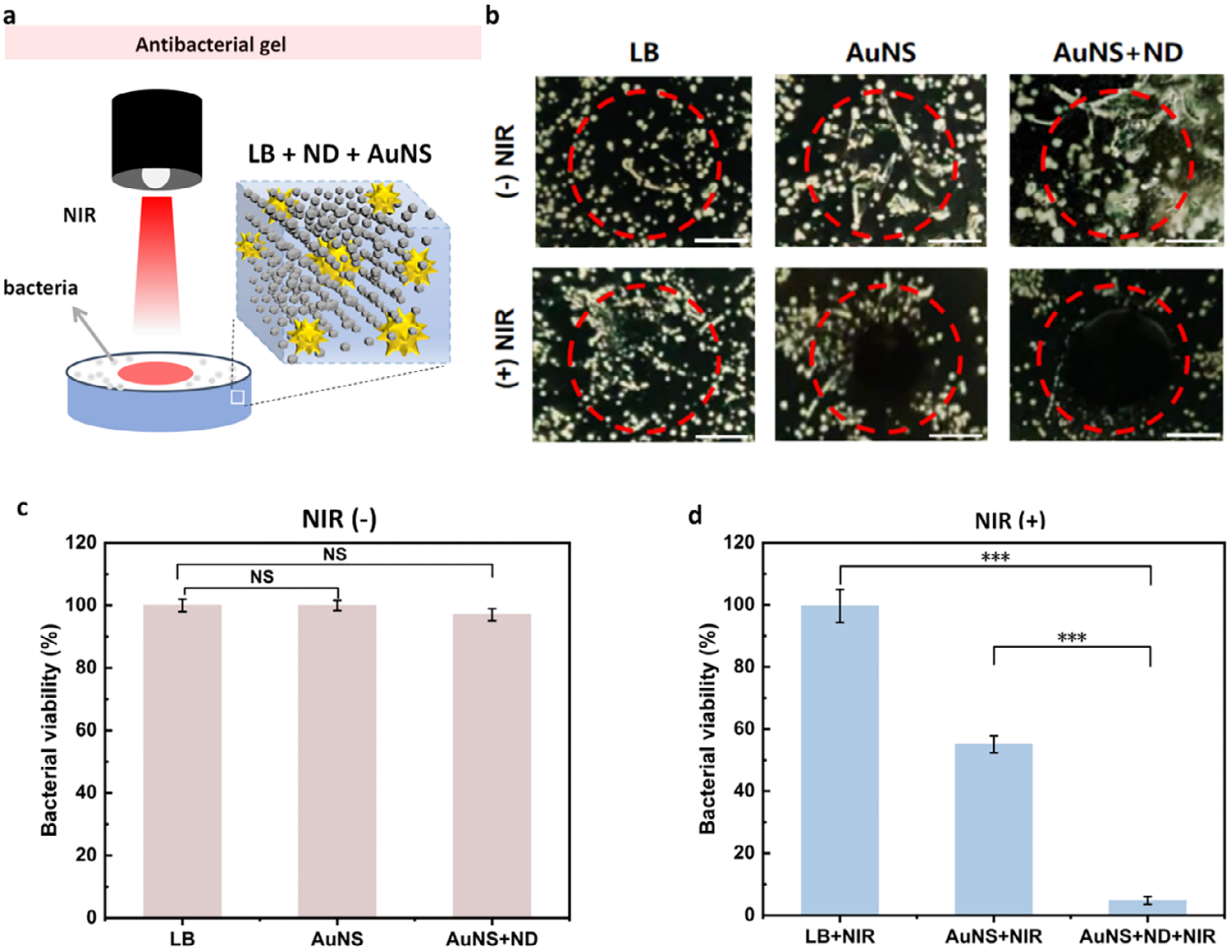
Synergistic photothermal eradication of *Staphylococcus aureus*. AuNS: 3.6 pM, ND: 3 × 10^−3^ v%. Time: 15 min, Power: 500 mW, Height: 10 cm. Incubation time: 30 min. (a) Schematic of the colony‐forming‐unit (CFU) assay used to evaluate antibacterial activity. LB agar is supplemented with nanoparticles, inoculated with *S. aureus*, pre‐incubated 2 h, and then exposed to an 808 nm laser (irradiated zone outlined in red). (b) Representative photographs of CFU plates after overnight incubation (37°C). Top row: control (no NIR), AuNS only (no NIR), AuNS + ND (no NIR). Bottom row: NIR‐irradiated counterparts showing the effect of photothermal treatment. Scale bars: 5 mm. (c) Statistical chart of bacterial viability for three groups without NIR (n = 3). (d) Statistical chart of bacterial viability for three groups with NIR (n = 3). Data are mean ± SD. One way ANOVA with Tukey's test. ^***^ *p* < 0.001; NS, no significant difference.

To test this idea, autoclaved LB agar was supplemented with three formulations: no particles, AuNS alone (3.6 pM), and AuNS + ND (AuNS: 3.6 pM, ND: 3 × 10^−3^ v%). After solidification, the plates were inoculated with *S. aureus* and pre‐incubated at 37°C for 2 h. A circular region (∼15 mm in diameter) was then exposed to an 808 nm laser for 15 min (Figures 4a; S16), and the plates were returned to 37°C overnight.

NIR irradiation alone did not affect bacterial viability, and neither ND nor AuNS showed toxicity in the dark, confirming their intrinsic biocompatibility. In contrast, AuNS‐containing plates subjected to NIR displayed a clear inhibition zone with colony numbers reduced by roughly half relative to untreated controls (Figure 4b–d, S17). The synergistic effect became striking when both nanoparticles were present: the AuNS + ND formulation under NIR left less than 5% colonies (most located at the circle edge) within the irradiated area, lowering the survival rate to about one‐tenth of that observed for AuNS + NIR. These results indicate that ND‐mediated photon trapping raises the local photon density available to nearby AuNS, which then convert the additional photons into heat, thereby amplifying the photothermal bactericidal effect beyond the additive sum of the individual components.

### 2.5 Endocytosis‐Driven Aggregates

**FIGURE 5:**
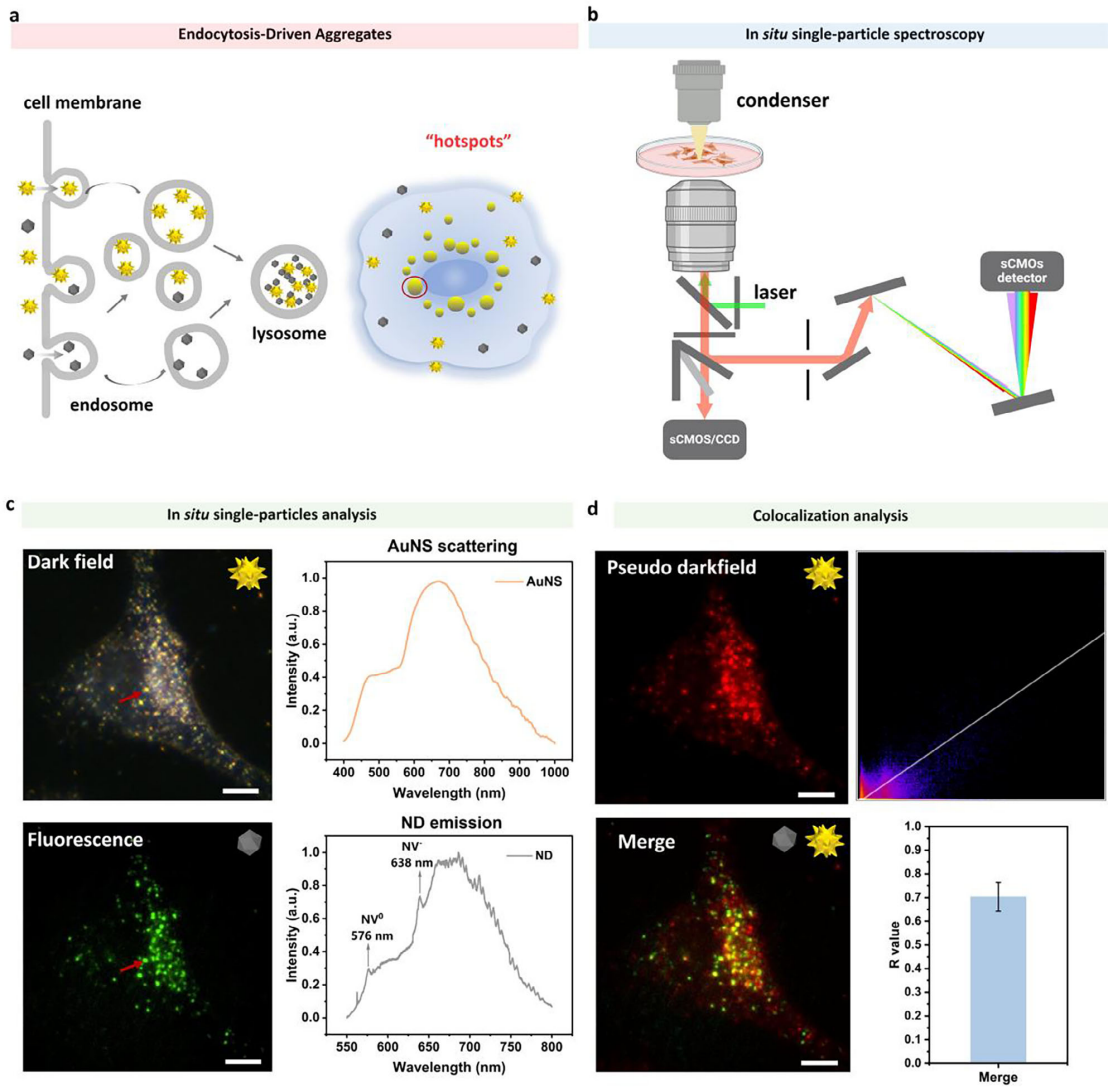
Intracellular co‐localisation of gold nanostars (AuNS) and nitrogen‐vacancy nanodiamonds (ND). AuNS: 1.8pM, ND: 7.5 × 10^−4^ v%. Incubation time: 12 h, overnight. (a) Conceptual sketch of a HeLa cell that has internalised both AuNS (yellow stars) and fluorescent ND (grey spheres). After endocytic trafficking, the two particle types converge and aggregate in late endosomes/lysosomes, forming a nanoscale photothermal “hotspot”. (b) Optical layout of the in situ spectroscopy platform. The same sub‐cellular region is sequentially interrogated in dark‐field (AuNS scattering) and TIRF fluorescence (ND emission); the scattering or fluorescence signal is routed to an sCMOS camera for imaging and, through a slit, to a spectrograph for single‐particle spectral read‐out. (c) In situ single‐particle analysis. Top‐left: dark‐field image of a representative cell (scale bar: 10 µm); the scattering spectrum from the red‐arrow region displays the broad AuNS LSPR band (∼ 650–800 nm). Bottom‐left: NV‐ND fluorescence image (scale bar: 10 µm); the emission spectrum from the white‐circled region shows the characteristic NV bands (∼ 575 nm and 638 nm) superimposed on a broad tail. (d) Pseudo‐colour composite of AuNS scattering (red) and ND fluorescence (green) reveals extensive yellow overlap, indicating spatial co‐localisation (scale bar: 10 µm). Right: scatter plot of pixel intensities and average Pearson correlation coefficient (r ∼ 0.70 ± 0.05, n = 3), confirming that the two optical signatures arise from the same intracellular hotspots.

Achieving intracellular photothermal ablation is pivotal for eradicating deeply seated tumor cells that escape conventional therapies and for minimizing systemic drug toxicity. Nanoparticles typically enter mammalian cells via receptor‐mediated endocytosis or by macropinocytosis; regardless of the entry route, most nanoparticles ultimately traffic to late endosomes or lysosomes [45, 46, 47], leading to their accumulation in a confined area. To translate the gel‐phase synergy of AuNS and ND into a cellular context, we first asked whether both nanoparticles would co‐accumulate in the same sub‐cellular compartment to form “hotspots” after uptake, thereby reproducing the coupled scattering‐and‐absorption mechanism inside living cells (Figure 5a).

To visualize this process unambiguously, ND were purpose‐engineered with nitrogen‐vacancy (NV) color centers that emit bright, photostable fluorescence, while AuNS provide a strong red‐shifted localized surface‐plasmon scattering signal under dark‐field illumination. The two nanoparticle types were co‐incubated with HeLa cells (AuNS ∼ 1.8 pM; ND ∼ 7.5 × 10^−^^4^ v%) for 12 h and imaged by an in situ spectroscopy platform (optical path in Figure 5b) that records AuNS dark‐field scattering, ND fluorescence, and their respective spectra from exactly the same sub‐micron region.

Using this set‐up, dense scattering puncta from AuNS and bright NV fluorescence from ND were both observed in the perinuclear zone that corresponds to the late endosome/lysosome population (Figures 5c; S18 and S19). Spectra collected at the red arrow‐marked spot confirmed that the broad AuNS LSPR band (∼650–800 nm) and the characteristic NV emission band of ND (∼550–750 nm) were detected simultaneously from the same spatial pixel (Figures 5c; S20), proving that the two nanoparticles occupy an identical nanoscale locale. When the scattering and fluorescence channels were merged (AuNS pseudo‐colored red, ND green; Figure 5d), extensive yellow overlap became apparent, and quantitative Pearson analysis returned a high co‐localization coefficient (R ∼ 0.70), far above the random‐overlap threshold.

In parallel, to validate the co‐localization of AuNS and ND within lysosomes, we performed lysosomal staining using Lyso‐Tracker. The results of three‐color co‐localization assays unambiguously revealed (Figure S21) that the dark‐field scattering signal of AuNS (pseudocolored red), the NV center fluorescence signal of ND (pseudocolored blue), and the lysosomal signal of LysoTracker (green) highly overlapped in the same subcellular region of the cells. Further quantitative colocalization analysis provided conclusive data for this observation: AuNS signals were detectable in approximately 79.3% of LysoTracker‐positive lysosomal structures, and ND signals were detectable in approximately 89.6% of lysosomes. These high colocalization ratios strongly demonstrate that endocytosed AuNS and ND indeed co‐localize in lysosomes.

These data establish that AuNS and fluorescent ND are co‐internalized and confined within individual endo‐lysosomal vesicles, where their intrinsic tendency to aggregate yields tightly packed hotspots; such intracellular architecture mirrors the physical proximity achieved in gels and is expected to couple ND‐mediated photon trapping with AuNS absorption, amplifying local photothermal conversion and providing the cellular basis for low‐dose photothermal ablation.

### 2.6 Low‐Power Photothermal Ablation

**FIGURE 6:**
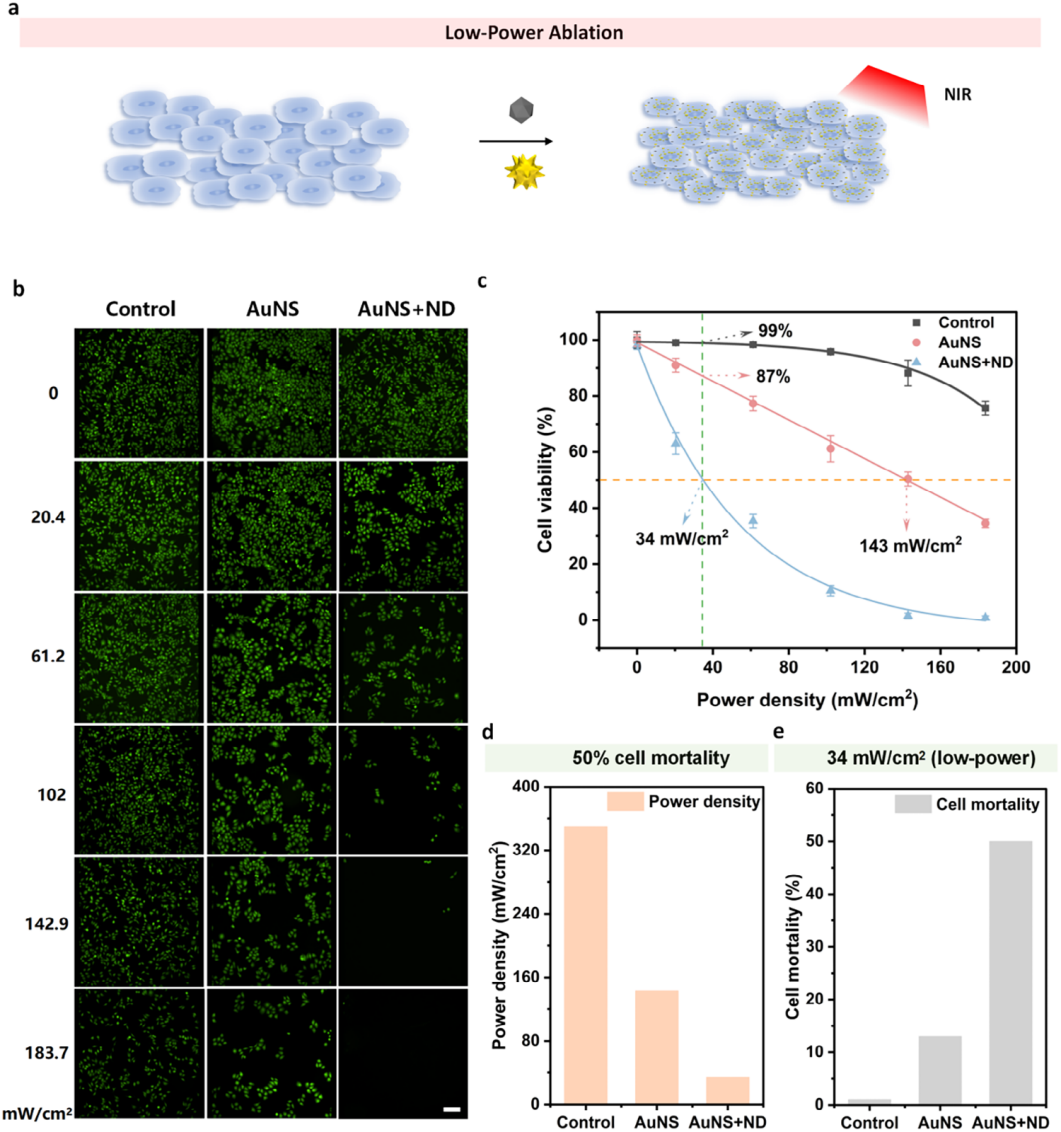
Low‐power photothermal ablation enabled by co‐internalised AuNS and ND. AuNS: 1.8 pM, ND: 7.5 × 10^−4^ v%. Time: 16 min, Power density: 0–183.7 mW/cm^2^, Height: 15 cm. Incubation time: 12 h, overnight. (a) Schematic diagram of HeLa cells endocytosing both AuNS and ND with near‐infrared light irradiation treatment. (b) Representative Calcein‐AM fluorescence micrographs (live cells appear green) of HeLa cultures containing no nanoparticles (Control), AuNS only, or AuNS + ND after 6 min of 808 nm irradiation (Height: 15 cm) at the indicated powers. Scale bars, 100 µm. (c) Cell‐viability curves for each formulation as a function of laser power density; data are mean ± SD (n = 3) and solid lines are sigmoidal fits. (d) Laser power density required to reduce viability to 50% (LD_50_) extracted from the fits. (e) Cell‐mortality percentages for all groups at a clinically relevant low dose of 34 mW cm^−^^2^, highlighting the markedly higher killing efficiency of the AuNS + ND formulation.

To test whether the intracellular hotspots formed by co‐internalised AuNS and ND translate into superior tumor‐cell killing, we compared three treatment groups—control (no particles), AuNS only (1.8 pM), and AuNS + ND (AuNS: 1.8 pM, ND: 7.5 × 10^−4^ v%) —under stepwise 808 nm irradiation. After overnight nanoparticle incubation, HeLa cultures were exposed for 6 min at total laser powers of 0, 100, 300, 500, 700, or 900 mW, corresponding to power densities of 0, 20.4, 61.2, 102.0, 142.9, and 183.7 mW cm^−^^2^ over the 2.5 cm‐diameter beam area. Cells were returned to 37°C for 4 h, stained with Calcein‐AM, and imaged to quantify viability (Figure 6a, b). To exclude bulk overheating as the cause of enhanced cytotoxicity, we continuously monitored the bulk temperature of the culture medium during NIR irradiation for the AuNS+ND group. As shown in Figure S22, even at the highest power density (183.7 mW·cm^−^^2^) for 300 s, the maximum temperature rise of the medium remained below 1°C, indicating negligible global overheating under our experimental conditions. These results support that the enhanced cell killing is primarily associated with localized intracellular photothermal hotspots rather than macroscopic medium heating.

At every dose, fluorescence weakened fastest in the AuNS + ND cohort, indicating the steepest loss of viability. Quantitatively, viability in this group plunged to ∼10% at 102 mW cm^−^^2^ and approached 0% at 143 mW cm^−^^2^. In contrast, AuNS alone required 183.7 mW cm^−^^2^ to reduce viability to ∼30%, while control cells retained >60% viability across the entire range. All groups remained ∼100% viable at 0 mW, confirming the intrinsic biocompatibility of both nanoparticles.

From the sigmoidal dose‐response curves, we extracted the power density that yields 50% mortality (Figure 6c). Co‐treated cells reached this threshold at only 34vs. 143 mW cm^−^^2^ for AuNS alone and an extrapolated 350 mW cm^−^^2^ for the control. Thus, ND lowers the effective dose by ∼ 4.2‐fold relative to AuNS alone and ∼10‐fold relative to untreated cells. Importantly, at 34 mW cm^−^^2^ the AuNS‐only group exhibited <15% mortality and the control group showed negligible death (Figure 6d), indicating that this low dose is benign to unsensitised cells while being lethal to hotspot‐bearing ones.

These findings support the mechanistic model established earlier: after endocytic uptake, AuNS and ND co‐aggregate in late endosomes or lysosomes. Upon NIR exposure, ND scatter and trap photons inside the confined vesicle, boosting local photon density; the neighbouring AuNS then absorb this enriched flux and convert it into heat with high efficiency. Compared with AuNS alone, the coupled ND + AuNS system harvests NIR energy far more effectively, enabling robust photothermal ablation at clinically safe, low‐power densities and offering a promising route to selective tumour therapy with minimal collateral damage.

### 2.7 In Vivo Antitumor Efficacy and Mechanism Validation

To evaluate the translational potential of this strategy into biologically relevant scenarios and verify its cooperative mechanism in a complex biological environment, we performed a comprehensive in vivo study using a xenograft tumor model. Subcutaneous tumors were established in immunodeficient BALB/c nude mice by inoculation with human Ramos B‐cell lymphoma cells. When tumor volumes reached 50–100 mm^3^, the mice were randomly divided into four groups, and intratumoral injections were administered as follows:(1) PBS group (50 µL per 100 mm^3^ tumor volume);(2) ND group (ND: 10 mg/mL, 10 µL per 100 mm^3^ tumor volume);(3) AuNS group (AuNS: 200 pM, 50 µL per 100 mm^3^ tumor volume);(4) AuNS+ND group (mixture with identical doses as the AuNS and ND single groups). Twenty‐four hours post‐injection, tumor regions were irradiated with an 808 nm laser at a power density of 500 mW/cm^2^ for 6 min. This treatment was repeated once daily for 5 consecutive days.

**FIGURE 7:**
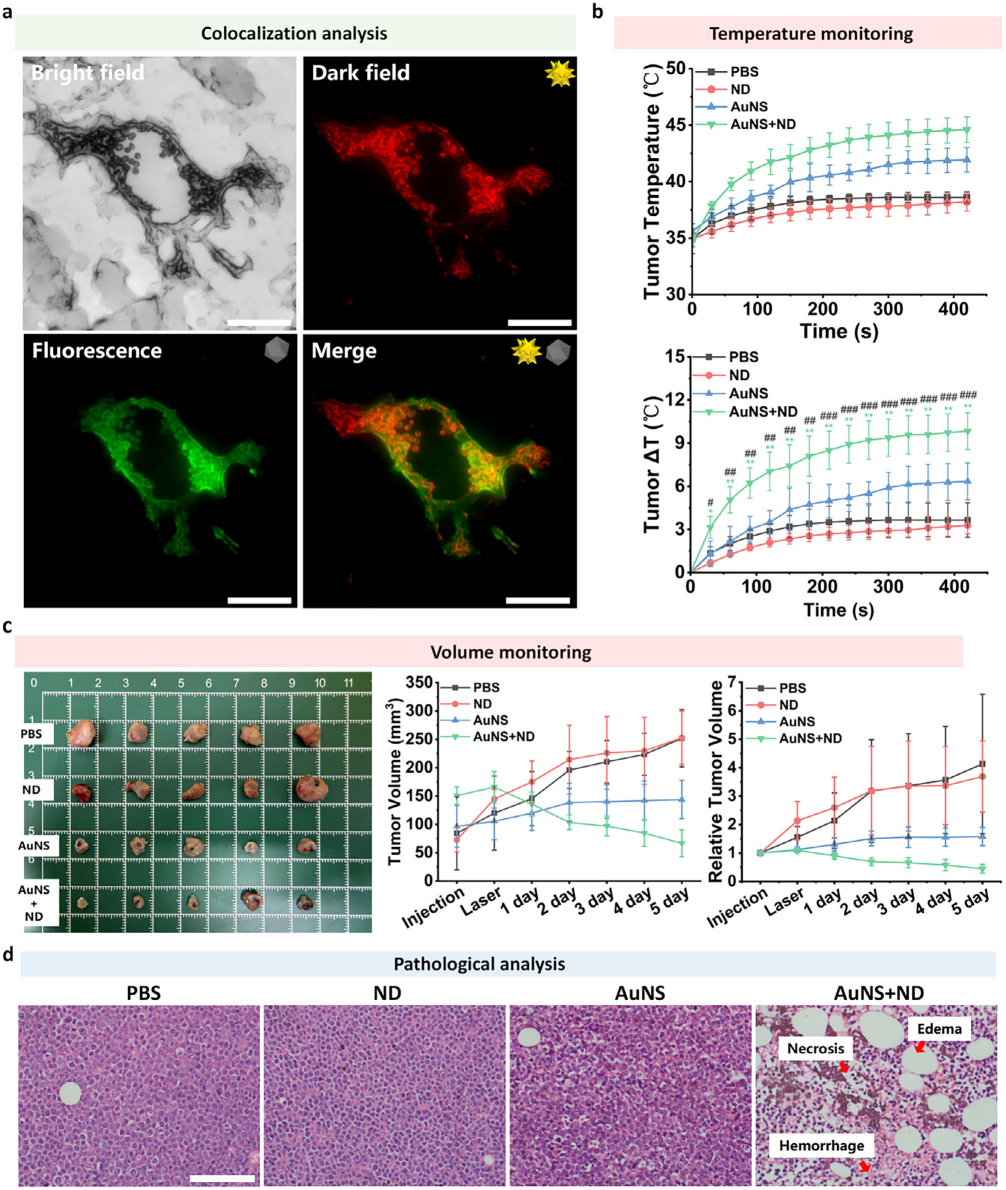
In Vivo Translation of the AuNS–ND Synergistic Effect. AuNS: 200pM, 50 µL/mm^3^. ND: 10 mg/mL, 10 µL/mm^3^. (a) Representative tumor cryosection images acquired 24 h after intratumoral administration of AuNS and fluorescent ND, showing co‐localization by bright‐field, dark‐field scattering of AuNS (pseudo‐colored red), ND fluorescence (pseudo‐colored green), and the merged image.Scale bars: 50 µm. (b) Real‐time monitoring of intratumoral core temperature (top) and the corresponding temperature rise (ΔT) (bottom) during 808 nm laser irradiation at 500 mW/cm^2^ for 6 min for different groups (PBS, ND, AuNS, and AuNS+ND). (c) Real‐time monitoring of intratumoral core temperature (top) and the corresponding temperature rise (ΔT) (bottom) during 808 nm laser irradiation at 500 mW·cm^−^^2^ for 6 min for different groups (PBS, ND, AuNS, and AuNS+ND). (d) Representative photographs of excised tumors (after the treatment period) and longitudinal monitoring of tumor volume and relative tumor volume (Vt/V0) over the five‐day treatment regimen (one irradiation per day). Tumor volume was calculated as V = 0.5 × L × W^2^. Data are mean ± SD (n = 5). Scale bar: 100 µm. One way ANOVA with Tukey's test. ^*^: AuNS+ND vs. AuNS. #: AuNS+ND vs. PBS. ^*^ *p* < 0.05, ^**^ *p* < 0.01, ^***^ *p* < 0.001. # *p* < 0.05, ## *p* < 0.01, ### *p* < 0.001.

First, to confirm that the cooperative mechanism remains operative in vivo, we employed nanodiamonds containing nitrogen‐vacancy (NV) color centers to examine the intratumoral distribution of the two nanomaterials. Cryosection imaging was performed on tumor tissues harvested 24 h after injection. The results showed distinct co‐localization of AuNS and ND signals within the tumor parenchyma in the same field of view (Figure 7a), and Pearson analysis further confirmed a high co‐localization coefficient of approximately 0.65 (Figure S23). These data verify that the two nanoparticles achieve precise co‐localization at the target site, fulfilling the spatial prerequisite for their near‐field coupling interaction.

With intratumoral co‐localization confirmed, we next evaluated the in vivo photothermal performance of the system. During the first laser irradiation session, real‐time temperature monitoring inside the tumor was conducted using a fine‐needle thermocouple. As shown in Figure 7b, tumors in the AuNS+ND group exhibited a significantly stronger and faster temperature increase, reaching a maximum temperature of 45°C with a maximum temperature elevation (ΔT) of approximately 9.5°C. In comparison, the AuNS‐only group showed a temperature rise of only ∼6°C, while the PBS and ND groups displayed negligible heating. These direct temperature measurements confirm that the ND‐mediated photon‐trapping effect remains functionally effective in vivo, thereby markedly enhancing the photothermal conversion efficiency of adjacent AuNS.

This enhanced photothermal effect translated into substantially improved therapeutic outcomes. Tumor growth was monitored continuously throughout the treatment period, and tumor tissues were excised for direct observation on the final day. As presented in Figures 7c and S24, the AuNS+ND group achieved the most pronounced tumor suppression, with tumor volumes progressively decreasing during treatment. The relative tumor volume in this combination group was significantly lower than that in all other groups, and the corresponding tumor control rate, a metric for tumor inhibition efficacy, was calculated to be approximately 75% (Figure S25). In contrast, tumors in the PBS and ND‐only groups grew rapidly, whereas the AuNS‐only group only induced moderate tumor growth delay. No obvious body weight loss was observed in any group throughout the experiment (Figure S26), indicating favorable biocompatibility of the hybrid system.

Hematoxylin and eosin (H&E) staining was performed on tumor tissues harvested 5 days after the first treatment to further characterize the therapeutic effect (Figures 7d; S27). Tumors in the AuNS+ND group exhibited extensive necrosis accompanied by apparent hemorrhage and interstitial edema, consistent with the hallmarks of effective photothermal ablation. In comparison, tumors from the AuNS‐only group displayed only focal damage, while those from the PBS and ND groups maintained the typical histological morphology of viable lymphoma cells.

In summary, the in vivo results establish a complete and consistent chain of evidence: the AuNS–ND hybrid strategy first enables intratumoral co‐localization of the two nanoparticles, which in turn generates significantly enhanced local photothermal heating, and ultimately achieves superior tumor suppression over single‐component treatments without systemic toxicity. This proof‐of‐concept validation in a living tumor model demonstrates that the scattering–absorption coupling principle represents a promising and viable approach for the development of low‐power, high‐precision photothermal therapeutic platforms.

## 3 Conclusions

Through rational coupling of gold nanostars (AuNS) with nanodiamonds (ND), we demonstrate a photon‐management strategy that overcomes the energy‐efficiency limits of conventional photothermal systems. By integrating FDTD nanoscale field mapping with mesoscopic Monte Carlo transport, we establish a causal chain in which ND‐mediated photon confinement extends the optical path length, elevates local photon density, and increases the absorbed power of adjacent AuNS, and we then validate this mechanism experimentally. Co‐embedded ND/AuNS gels exhibit about 90% larger temperature rises than AuNS alone, in quantitative agreement with the simulated photon‐density gains, and this enhanced heating underpins potent photothermal sterilization. In cells, endocytosis drives AuNS and ND into lysosomal “hotspots” that reproduce the modeled conditions, lowering the LD50 for tumor‐cell ablation by approximately 4.2‐fold under clinically relevant irradiance. Importantly, the ability to lower the required NIR irradiance is not merely a performance metric but a safety‐enabling advantage. In biomedical settings, the practical limit of photothermal therapy is often set by off‐target heating in non‐labeled regions rather than by the absorber itself. By using NDs as biocompatible, broadband passive photon managers (with negligible intrinsic absorption), our approach increases photon availability specifically where scatterers and absorbers co‐localize, thereby strengthening localized hotspot formation while mitigating unnecessary heating elsewhere under the same incident conditions. This provides an orthogonal route to improving photothermal action beyond conventional strategies that rely on higher absorber doses or newly engineered high‐absorption materials—both of which face constraints in biocompatibility and translational tractability. ND functions as passive optical amplifiers with negligible intrinsic absorption, explaining how trace additives can deliver large efficiency gains.

Because the scattering‐plus‐absorption concept is modular, any broadband scatterer can in principle be paired with any efficient absorber to tune operating wavelength, heating rate, or imaging readout. The color‐center optics of ND further enable self‐reporting or feedback‐controlled phototherapies. Beyond proof‐of‐principle demonstrations in gels, bacteria, and cultured tumor cells, this coupling strategy offers a versatile platform for oncotherapy, postoperative sterilization, and other light‐managed biomedical or catalytic processes where energy efficiency is paramount. By showing how a simple geometric arrangement unlocks low‐dose yet high‐potency photothermal action, this work lays the groundwork for photonic nanomedicines and broader light‐responsive technologies.

## 4 Materials and Methods

Supporting Information: Section S1 – S21.

## Author Contributions

The manuscript was written through the contributions of all authors. All authors have given approval to the final version of the manuscript.

## Conflicts of Interest

The authors declare no conflicts of interest.

## Supporting information




**Supporting File**: advs74634‐sup‐0001‐SuppMat.docx.

## Data Availability

The data that support the findings of this study are available in the supplementary material of this article.
