# Supplementary material for: Light‐Harvesting Photothermal Hotspots Enabled by NIR Scattering‐Absorption Coupling
Source: Adv Sci (Weinh). 2026 Mar 9;13(27):e23721. doi: 10.1002/advs.202523721 (PMC13170250; doi:10.1002/advs.202523721)
Supplement: Supplementary file 1 — Supporting File: advs74634‐sup‐0001‐SuppMat.docx. [file ADVS-13-e23721-s001.docx]

Supporting Information

**Light-Harvesting Photothermal Hotspots Enabled by NIR Scattering-Absorption Coupling**

Zebin Wu ^a #^, Changming Bao ^a #^, Pengyu Zhang ^b #^, Tao Ding ^a^, Weiming Lin ^a^, Haodong Li ^a^, Wenjian Luo ^a^, Xizhi Deng ^a^, Hao Wang ^c^, Guanle Li ^b^, Hongyue Hu ^a^, Ye Wang ^a^, Dawei Jiang ^c^, Yu Zhang ^d^, Sisi Jia ^e^, Nan Zhang ^b^ *，Min Ke ^b^ * and Le Liang ^a^ *

^a^ The Institute for Advanced Studies (IAS) for Wuhan University, Department of Ophthalmology, Zhongnan Hospital of Wuhan University, State Key Laboratory of Metabolism and Regulation in Complex Organisms, College of Life Sciences, Wuhan University, Wuhan 430072, China.

^b^ Department of Ophthalmology, Zhongnan Hospital of Wuhan University, Wuhan 430071, China.

^c^ Department of Nuclear Medicine, Union Hospital, Tongji Medical College, Huazhong University of Science and Technology, Wuhan, 430022, China

^d^ The Interdisciplinary Research Center, Shanghai Synchrotron Radiation Facility, Zhangjiang Laboratory, Shanghai Advanced Research Institute, Chinese Academy of Sciences, Shanghai 201210, China

^e^ Zhangjiang Laboratory, Shanghai 201210, China.

^#^ These authors contributed equally to this work.

* Corresponding authors:

le.liang@whu.edu.cn

kemin@whu.edu.cn

nan.zhang@whu.edu.cn

**Multiphysics Modeling Methodology**

To systematically evaluate the photothermal conversion behavior and temperature rise characteristics of gold nanomaterials under near-infrared laser irradiation, we established a comprehensive multiphysics simulation workflow integrating electromagnetic field simulation, Monte Carlo photon transport modeling, and thermal diffusion analysis. The modeling methods and parameter settings for each submodule are detailed below.

**S1 FDTD Electromagnetic Simulation**

Three-dimensional finite-difference time-domain (FDTD) simulations were performed using Lumerical FDTD Solutions (Ansys 2020 R2). The gold nanostars (AuNS) were modeled as nanostructures with 20 protruding spikes, with an overall diameter of 70 nm. Nanodiamonds (ND) were represented as regular icosahedra with a diameter of 10 nm and refractive index n = 2.4173. ND were uniformly distributed around the AuNS surface and near the spike tips within a spherical shell domain, at a volumetric density of 6.11*10^-6^ particles/nm³ to replicate experimental spatial distributions.

The simulation domain was filled with a water medium (n = 1.33), and perfectly matched layer (PML) boundary conditions were applied in all directions to suppress reflections. A total-field scattered-field (TFSF) plane wave source was used, incident along the long axis of the AuNS, with a center wavelength of 808 nm. The simulation time was set sufficiently long to ensure the complete decay of transient fields and allow for the extraction of steady-state responses.

Built-in monitors were placed inside and outside the TFSF source to record absorption (σ_abs_) and scattering (σ_sca_) cross-sections. All optical responses were normalized to those of a single AuNS to assess the effect of ND surface modification on local field enhancement. A nonuniform adaptive mesh was employed, with the minimum mesh size <2 nm to accurately resolve the strong near-field gradients around the nanospikes. From the obtained absorption cross-section σ_abs_​ and scattering cross-section σ_sca​_, we calculated the absorption coefficient μ_a_​ and scattering coefficient μ_s_​. The relationships are given by μ_a_​=Nσ_abs_​ and μ_s_​=Nσ_sca_​, where N denotes the particle number density.

Structural modeling and ND spatial configuration were completed using 3ds Max and imported into the FDTD environment for simulation.

**S1.1 Explanation for the Non-Absorbing Nature of ND**

In the Supplementary Information, ND has diameter 10nm (radius r=5nm). For illumination at λ0=808 nm in a medium with refractive index $n_{m}$≈1.37, the size parameter is:

$$x=\frac{2\pi n_{m}r}{\lambda_{0}}$$

which satisfies x≪1, indicating that NDs are in the Rayleigh limit. Therefore, instead of the full Mie series, we used the Rayleigh approximations for the scattering and absorption cross sections:

$$C_{\mathrm{sca}}=\frac{8\pi}{3}k_{m}^{4}r^{6}\left| \frac{m^{2}-1}{m^{2}+2} \right| C_{\mathrm{abs}}=4\pi k_{m}r^{3}Im(\frac{m^{2}-1}{m^{2}+2})$$

where $k_{m}=2\pi n_{m}/\lambda_{0}$ is the wavenumber in the medium and

$$m=\frac{n_{p}+ik_{p}}{n_{m}}.$$

is the complex refractive-index ratio (particle to medium), with $\text{np​+i}\text{k}_{\text{p}}$ ​the complex refractive index of ND. Using the optical constants of ND at 808 nm ($\text{n}_{\text{p}}\text{​=2.4044, }\text{k}_{\text{p}}\text{≈0}$), the absorption term is negligible, i.e.,$\text{C}_{\text{abs}}\text{≈0}$, so ND mainly contributes to scattering (and thus to $\text{μ}_{\text{s}}$ or $\text{μ}_{\text{s}}^{\text{'}}$ ​), while its contribution to $\mu_{a}$​ can be neglected or treated as a secondary term.

**S1.2 Distance-parameterized AuNS–ND separation analysis**

To address the influence of inter-component distance on optical absorption, we performed an additional distance-parameterized analysis using Lumerical FDTD Solutions. The key principle is to keep all geometry, materials, and solver settings fixed and vary only the AuNS–ND separation distance, then compute the absorption cross section of the coupled system and normalize it to the absorption cross section of AuNS alone. This yields an absorption enhancement factor as a function of distance.

**S1.2.1 Distance definition and scan points**

The separation distance $d$ is defined as the shortest distance from the ND center to the AuNS surface (center-to-surface distance).For each scan point, the ND was repositioned such that this center-to-surface distance equals $d$, while all other parameters remained unchanged.

A non-uniform distance scan was used to cover both weak-coupling (far-field) and strong-coupling (near-field) regimes.The scanned distances (in nm) were:

$d\in\{$2500, 2000, 1600, 1300, 1000, 800, 600, 450, 350, 270, 200, 150, 120, 100, 80, 60, 50, 30, 20, 10$\}$

**S1.2.2 FDTD model settings for the distance scan**

All FDTD settings not explicitly listed below were kept identical to those in Section S1, including particle geometry, background medium, boundary conditions, excitation configuration, and normalization strategy.

For completeness, the key items specific to this scan were:

- **Simulation type:** 3D FDTD.
- **Background medium:** water (consistent with S1).
- **Materials:** AuNS material = *Au (Gold) – CRC* (built-in); ND refractive index fixed at $n=2.4173$.
- **Mesh:** auto non-uniform; mesh accuracy = 2; minimum mesh step = 2.5 nm (used for this scan).
- **Boundaries:** PML on all six boundaries (x/y/z min/max).
- **Source:** TFSF source (same type as used for cross-section extraction in S1).

**S1.2.3 Absorption cross section extraction**

The absorption-related cross section was obtained using the built-in Optical Power → Cross Section workflow in Lumerical, which constructs a closed surface around the target structure using power monitors and evaluates the net outward power flow to obtain the cross section spectrum. Briefly, six 2D power monitors were placed on the faces of an enclosing box $(x_{1}/x_{2}, y_{1}/y_{2},z_{1}/z_{2}z2)$ to form a closed surface around the structure. The transmitted power through each monitor was obtained and summed over all six faces to compute the total outward power flow $P_{out}(\lambda)$. The absorption cross section spectrum was then computed by normalizing the total outward power by the incident source intensity$I_{inc}\left( \lambda\right)$ using the built-in source normalization, i.e.,

$$\sigma_{abs}(\lambda)=\frac{P_{out}(\lambda)}{I_{inc}(\lambda)}.$$

This procedure was applied consistently to both the coupled system (AuNS+ND) and the reference AuNS-alone simulation under otherwise identical settings.

**S1.2.4 Enhancement factor definition and distance dependence**

For each distance $d$, the absorption enhancement factor was defined as the ratio of the absorption cross section of the coupled system to that of AuNS alone:

$$EF(d)=\left. \frac{\sigma_{abs}^{AuNS+ND}(d)}{\sigma_{abs}^{AuNS}} \right|_{\lambda=808 nm}.$$

This normalization isolates the distance-dependent coupling effect by using the AuNS-alone response as a reference under the same simulation configuration.

The resulting $EF\left( d \right)$ curve is summarized in *Figure S1*.

**S2 Monte Carlo Photon Transport Simulation (Photon Absorption and Photon Density)**

To evaluate the spatial light distribution and heat absorption characteristics in cylindrical phantoms under 808 nm laser illumination, three-dimensional Monte Carlo photon transport simulations were performed using the GPU-accelerated MCXLAB toolkit. Four simulation scenarios were designed to analyze the effects of different nanoparticle combinations on light absorption and scattering:

• Case 1: Background medium only (no particles);

• Case 2: Gold nanostars (AuNS) only, considering inherent absorption and scattering;

• Case 3: Nanodiamond (ND) only, providing scattering;

• Case 4: AuNS + ND coexisting, exhibiting combined absorption and scattering.

Optical cross-sections of the particles were derived from the preceding FDTD results.

**S2.1 Optical and Geometric Parameters**

The phantom was a cylindrical gel with a diameter of 16 mm and height of 10 mm. The background medium had a refractive index n = 1.37, anisotropy factor g = 0.9, absorption coefficient μ_a_ = 0.007mm^-1^, and scattering coefficient μ_s_ = 0.02mm^-1^. The spatial resolution was set to 0.05 mm. A 2D circular mask was used to define the XY geometry and extruded along Z to form the 3D voxel model.

The AuNS absorption cross-section was set to 1.27*10^-14^m^2^ (raw) or 1.55*10^-14^ m^2^ (coupling-corrected), with a scattering cross-section of 1.59*10^-15^m^2^ and a concentration of 3.6 pM. The macroscopic scattering coefficient of ND was 0.0438mm^-1^. All optical parameters were converted to volume-scale coefficients (mm⁻¹) using Avogadro’s number.

All optical parameters used for Monte Carlo simulations at 808 nm and their sources are summarized in *Table S1*. Background parameters were taken from literature, whereas particle cross-sections were obtained from FDTD simulations. The macroscopic scattering coefficient of ND was calculated based on Mie scattering.

**S2.2 Light Source and Simulation Parameters**

A total of 10^10^ photons were launched from a source positioned 1 mm above the phantom, with perpendicular incidence along the -Z direction. The simulation time ranged from 0 to 5 ns with a timestep of 0.5 ns. All simulations ran on GPU #1 using the autopilot mode to optimize performance.

**S2.3 Absorbed Power Density Calculation**

After photon transport simulations, the steady-state photon flux distribution Φ(x, y, z) was calculated by summing time-resolved flux data at each voxel:

$$\Phi\left( x,y,z \right)=\sum_{t} data \left( x,y,z,t \right)$$

The absorbed power density Q_abs_ (x, y, z) was then calculated using the local absorption coefficient:

$$Q_{abs}(x,y,z)=\mu_{a}(x,y,z)\Phi(x,y,z)$$

**S2.4 Sensitivity Analysis of Optical Parameters**

To evaluate the robustness of the Monte Carlo (MC) photon-transport predictions with respect to uncertainties in optical parameters, we performed a systematic parametric sensitivity analysis by perturbing key optical parameters around their nominal values and quantifying the resulting change in the absorption-related output $A$.

**S2.4.1 Sensitivity definition and perturbation protocol**

For each optical parameter $p$, we performed a parametric sensitivity analysis by perturbing the key optical parameters

$$p\in\{\mu_{\alpha,bkg},\mu_{s,bkg},,g,n,\mu_{a,AuNS},\mu_{s,ND}\}$$

we applied a relative perturbation $\Delta p$ in the range of ±10% to ±50% while keeping all other parameters fixed, and recomputed the corresponding model output $A$. The normalized (relative) sensitivity coefficient was defined as

$$S_{p}=\frac{\Delta A/A_{0}}{\Delta p/p_{0}}=\frac{(A(p)-A_{0})/A_{0}}{(p-p_{0})/p_{0}}$$

which represents the relative change in the output per relative change in the parameter and can be interpreted as a finite-difference approximation of $\partial lnA/\partial lnp$. This definition enables a consistent comparison across parameters with different units and magnitudes.

**S2.4.2 Sensitivity classification criteria**

For clarity of discussion, we classified sensitivity based on $\mid S_{p}\mid$ using the following operational thresholds

- $\mid S_{p}\mid\leq0.1$: not sensitive
- $0.1<|S_{p}| <0.5$: moderately sensitive
- $|S_{p}|\geq0.5$: very sensitive

**S2.4.3 Global absorption sensitivity**

The sensitivity heatmap and the (|S|)-based category plot (*Figure S3*) summarize the dependence of the absorption-rate metric $A$ on the above parameters over the tested perturbation ranges. In general, $A$ is most sensitive to nanoparticle absorption-related parameter(s), moderately sensitive to $g$ and $n$, and comparatively weakly sensitive to the background optical coefficients $\mu_{a,bkg}$ and $\mu_{s,bkg}$, as well as to $\mu_{s,ND}$. This supports that the primary trends in absorption-rate outcomes are not dictated by the specific choice of background optical coefficients, but are driven by the nanoparticle-induced absorption increment, consistent with the intended use of the model as a controlled reference framework.

**S2.4.4 Auxiliary sensitivity analysis for spatial redistribution induced by ND scattering**

When the model output is defined as a global absorption-related quantity, varying the nanodiamond scattering coefficient $\mu_{s,ND}$​ produces only a limited change in the predicted outcome. Importantly, in light transport, scattering-related parameters often have a primary effect on the spatial redistribution of photon trajectories and fluence, rather than on the net absorbed energy integrated over the entire domain. Therefore, to explicitly quantify the redistribution effect induced by $\mu_{s,ND}$​, we conducted an auxiliary sensitivity analysis using an additional spatial metric defined around the optical axis (*Figure S4)*.

Specifically, we scaled $\mu_{s,ND}$ ​ around its nominal value $\mu_{s,ND}^{0}$ ​ with a multiplicative factor $\alpha$ in the range $\alpha=0.5-1.5$ (corresponding to −50% to +50% relative perturbations). For each $\alpha$, one MC simulation was performed to obtain the time-integrated three-dimensional fluence distribution $\Phi(x,y,z)$. The corresponding absorption-related field was computed as

$$Q_{abs}(x,y,z)=\mu_{a,total}(x,y,z)\Phi(x,y,z)$$

where $\mu_{a,total}$​ denotes the total absorption coefficient under the corresponding condition.

Based on $Q_{abs}(x,y,z)$, we extracted two complementary outputs:

1. **Global absorption (contrast reference):**

$$A_{global}=\iiint_{\Omega}Q_{abs}(x,y,z)dV$$

where $\Omega$ denotes the entire simulation domain.

1. **Redistribution metric (axial ROI energy fraction):**

$$F_{ROI}=\frac{\iiint_{\Omega_{ROI}}Q_{abs}(x,y,z)dV}{\iiint_{\Omega}Q_{abs}(x,y,z)dV},$$

where $\Omega_{ROI}$ is a cylindrical region-of-interest centered on the optical axis with radius $R_{ROI}$​ and depth range $[z_{1},z_{2}]$.

The sensitivity computation for $A_{global}$​ and $F_{ROI}$ follows the same normalized procedure described in S2.4.1. The resulting trends (*Figure S4*) illustrate that $\mu_{s,ND}$ ​ primarily affects the spatial deposition pattern (localization/spread of absorption) rather than the global absorption magnitude, thereby reconciling weak global sensitivity with a physically meaningful redistribution effect.

**S3. Three-Dimensional Monte Carlo Photon Transport Simulation (Photon Path)**

To systematically investigate light transport and scattering behaviors of metallic and dielectric nanoparticles embedded in tissue-mimicking gel, a custom-built 3D Monte Carlo photon transport algorithm was developed. The simulation geometry was defined as a cylindrical gel phantom (diameter = 16 mm, height = 10 mm), and four nanoparticle configurations were modeled:

• Case 1: Background medium only (no particles);

• Case 2: Gold nanostars (AuNS) only, considering inherent absorption and scattering;

• Case 3: Nanodiamond (ND) only, providing scattering;

• Case 4: AuNS + ND coexisting, exhibiting combined absorption and scattering.

For each case, 5*10^6^ photon packets were launched and their full transport trajectories were recorded.

**S3.1 Optical and Geometric Parameters**

The background hydrogel was assigned a refractive index of n = 1.33, absorption coefficient μₐ = 0.07 cm⁻^1^, scattering coefficient μₛ = 0.2 cm⁻^1^, and anisotropy factor g = 0.9. The optical cross-sections of AuNS were obtained from FDTD simulations as σ_abs_ = 1.27*10⁻^14^m² and σ_sca_ = 1.59 *10⁻^15^m², with a volumetric concentration of 3.6 pM. ND provided pure scattering with a macroscopic coefficient of μₛ = 0.4381 cm⁻¹. For the coupled case, enhanced absorption was modeled using an increased cross-section σ_abs_coupled_ = 1.55*10⁻^14^m². The simulation domain was discretized into a 400*400*400 voxel grid, with resolutions of dx = dy = 0.004 cm, dz = 0.0025 cm.

**S3.2 Photon Launching and Propagation Model**

Each photon was launched from near the upper edge of the cylinder and propagated downward along the -Z axis. The free path s was sampled from an exponential distribution:

$$s=-\frac{\ln\xi}{\mu_{t}}$$

$$\mu_{t}=\mu_{a}+\mu_{s}$$

$$\xi\sim u(0,1)$$

Absorption along each step was implemented continuously using:

$$\Delta E=w\cdot\left( 1-e^{-\mu_{a}\cdot s} \right)$$

where $w$ is the current photon weight. The scattering direction was sampled based on the Henyey–Greenstein phase function:

$$\cos\theta=\frac{1}{2g}\left[ 1+g^{2}-\left( \frac{1-g^{2}}{1-g+2g\xi} \right)^{2} \right]$$

All boundary surfaces (top, bottom, and cylindrical side wall) were modeled using Schlick’s approximation for Fresnel reflectance::

$$R(\theta)=R_{0}+(1-R_{0})(1-\cos\theta)^{5}$$

$$R_{0}=\left( \frac{n_{in}-n_{out}}{n_{in}+n_{out}} \right)^{2}$$

Upon reaching a boundary, photons were either reflected (with updated direction) or transmitted out of the medium. If the photon weight dropped below a threshold (1*10^-4^), a Russian roulette mechanism with a survival probability of 0.1 was applied to avoid excessive computation.

**S3.3 Pathlength and Energy Statistics**

Each photon’s total pathlength was recorded and used to generate histogram distributions. After completing all simulations, the absorbed energy fraction (A), transmitted (T), reflected (R), and side-escaped (S) fractions were computed and normalized to the total photon count.
For reference, the theoretical absorption under non-scattering conditions was calculated using the Beer–Lambert law::

$$A_{theory}=1-e^{-\mu_{a}\cdot d}$$

and the expected mean pathlength based on transport theory was given by:

$$\langle L\rangle_{theory}=\frac{1}{\mu_{a}+\mu_{s}\left( 1-g \right)}$$

All spatial and statistical results were saved in structured formats for downstream thermal modeling and validation against experimental measurements.

**S4 Gaussian Volumetric Thermal Diffusion Model and Experimental Calibration**

To model the thermal response of gold nanomaterials under near-infrared laser heating, a 2D Gaussian volumetric absorption model was used in combination with thermal diffusion theory. The temperature evolution is driven by the Monte Carlo (MC)–derived absorption rate (absorption fraction), together with a macroscopic heat-exchange correction and an experimental calibration step.

The thermal model assumptions are as follows: (i) the agarose gel phantom is treated as a homogeneous and isotropic effective medium; (ii) thermal properties are constant in time and space, including thermal conductivity $k$, density $\rho$, and heat capacity $c_{p}$​; (iii) no perfusion and no metabolic heat generation are considered, consistent with an in-vitro agarose phantom, and heat transfer inside the gel is dominated by conduction; (iv) the heat source is proportional to absorbed optical energy, where the absorbed fraction is obtained from MC simulations and converted into an effective absorbed power under laser irradiation. This model is intended for an in-vitro agarose phantom and neglects secondary effects (e.g., perfusion, evaporation, detailed container-contact heat transfer), which primarily impact absolute magnitude rather than the absorption-driven trends discussed here.

**S4.1 Parameter Settings and Source Modeling**

Laser parameters were: power $P=500 mW$, beam radius $w=120\mu m$, and photothermal conversion efficiency $\eta=0.45$. The ambient temperature was $T_{0}={25}^{\circ}C$. Absorption rate (denoted as ${abs}_{rate}$, i.e., absorption fraction) was obtained from simulations. Observation depth was $z_{m}=1 mm$; radial observation positions were set to $r=3,6,9,12 mm$.

supplementary information

Thermal properties were $k=0.6 W/m\cdot K$, $\rho=1000 kg/m^{3}$, and $c_{p}=4200 J/kg\cdot K,$ giving thermal diffusivity $\alpha=k/(\rho\cdot c_{p})$. These values were treated as constants throughout the phantom.

**S4.2 Optical-Thermal Model and Transient Temperature Solution**

**Optical-to-thermal source coupling**

The effective absorbed power at the observation depth zmz_mzm​ is modeled using Beer–Lambert attenuation:

$$P_{abs}=P\cdot{abs}_{rate}\cdot\eta\cdot exp(-\mu_{a}z_{m}),$$

where $\mu_{a}$ denotes the effective absorption coefficient along the optical axis consistent with the optical coefficients used in the MC model.

The radial deposition profile is weighted by a 2D Gaussian beam. The normalized Gaussian weight used to distribute the absorbed power across radial source shells is

$$f(r')=\frac{2}{w_{0}^{2}}r'exp[-2{(\frac{r'}{w_{0}})}^{2}].$$

**Transient temperature solution**

For an observation radius rrr (evaluated at $r=3,6,9,12 mm$ at depth $z_{m}$ ​), the transient temperature is obtained by integrating the 3D thermal diffusion Green’s function over all Gaussian-weighted source shells:

$$T(r,t)=T_{env}+\frac{P_{abs}}{\rho c_{p}}\int_{0}^{r_{max}} f(r')\frac{1}{4\pi kR}erfc(\frac{R}{2\sqrt{\alpha t}})dr' ,$$

where $erfc(\cdot)$ is the complementary error function and

$$R=\sqrt{{(r-r')}^{2}+z_{m}^{2}}.$$

In practice, the radial integration is truncated at the sample radius. Here we use $r_{max}=5 mm$ (10 mm diameter cuvette), matching the e

xperimental container size. This is the same structure implemented in the code (use of $f(r')$, the $1/(4\pi kR)$ factor, and numerical radial integration).

**S4.3 Macroscopic Heat Exchange Correction and Experimental Calibration**

To account for thermal exchange between the sample and environment (including unavoidable heat loss and measurement-position effects), a macroscopic heat-exchange correction using a time constant $\tau_{macro}$​ was introduced:

$$T_{smooth}(t)=T_{env}+(T(r,t)-T_{env})(1-e^{-t/\tau_{macro}}),$$

with

$$\tau_{macro}=240 s.$$

The macroscopic heat-exchange time constant $\tau_{macro}$ was obtained by fitting the baseline (blank gel) heating curve under identical irradiation conditions and was then fixed for all groups.

Finally, a time-invariant multiplicative calibration factor was applied to align the model magnitude with experimental readings:

$$correction factor(i)=\frac{\Delta T_{exp}(i)}{\Delta T_{sim}(i)}.$$

In practice, the correction factor is determined using the temperature rise at a fixed calibration time point (10 minutes) and the same sensor positions for all groups. We emphasize that the diffusion model, driven by the MC-derived absorbed power, determines the temporal evolution and relative comparison between conditions; the experimental calibration is applied only as a time-invariant multiplicative amplitude correction to account for systematic heat-loss and probe-position effects. The same calibration protocol was used for all groups, so no case-specific tuning degrees of freedom were introduced.

***Table S1***. All optical parameters used for Monte Carlo simulations at 808 nm and their sources

| Parameter | Value (at 808 nm) | Source |
| --- | --- | --- |
| g | 0.90 | Jacques (2013)^1^ |
| n | 1.37 | Jacques (2013)^1^ |
| $\mu_{a bkg}$ | 0.007 mm⁻¹ | Hale & Querry (1973)^2^ |
| $\mu_{s bkg}$ | 0.020 mm⁻¹ | Hale & Querry (1973)^2^ |
| $\mu_{s ND}$  $\mu_{a AuNS}$  $\mu_{s AuNS}$  $\mu_{a Coupling (AuNS+ND)}$ | 0.0438 mm⁻¹  0.0345 mm^-1^  0.0234 mm^-1^  0.0405 mm-^1^ | Calculated via Mie scattering  FDTD Simulation  FDTD Simulation  FDTD Simulation |
| $\sigma_{a AuNS}$ | 1.27 × 10⁻¹⁴ m² | FDTD Simulation |
| $\sigma_{s AuNS}$ | 1.59 × 10⁻¹⁵ m² | FDTD Simulation |
| $\sigma_{a Coupling (AuNS+ND)}$ | 1.55 × 10⁻¹⁴ m² | FDTD Simulation |


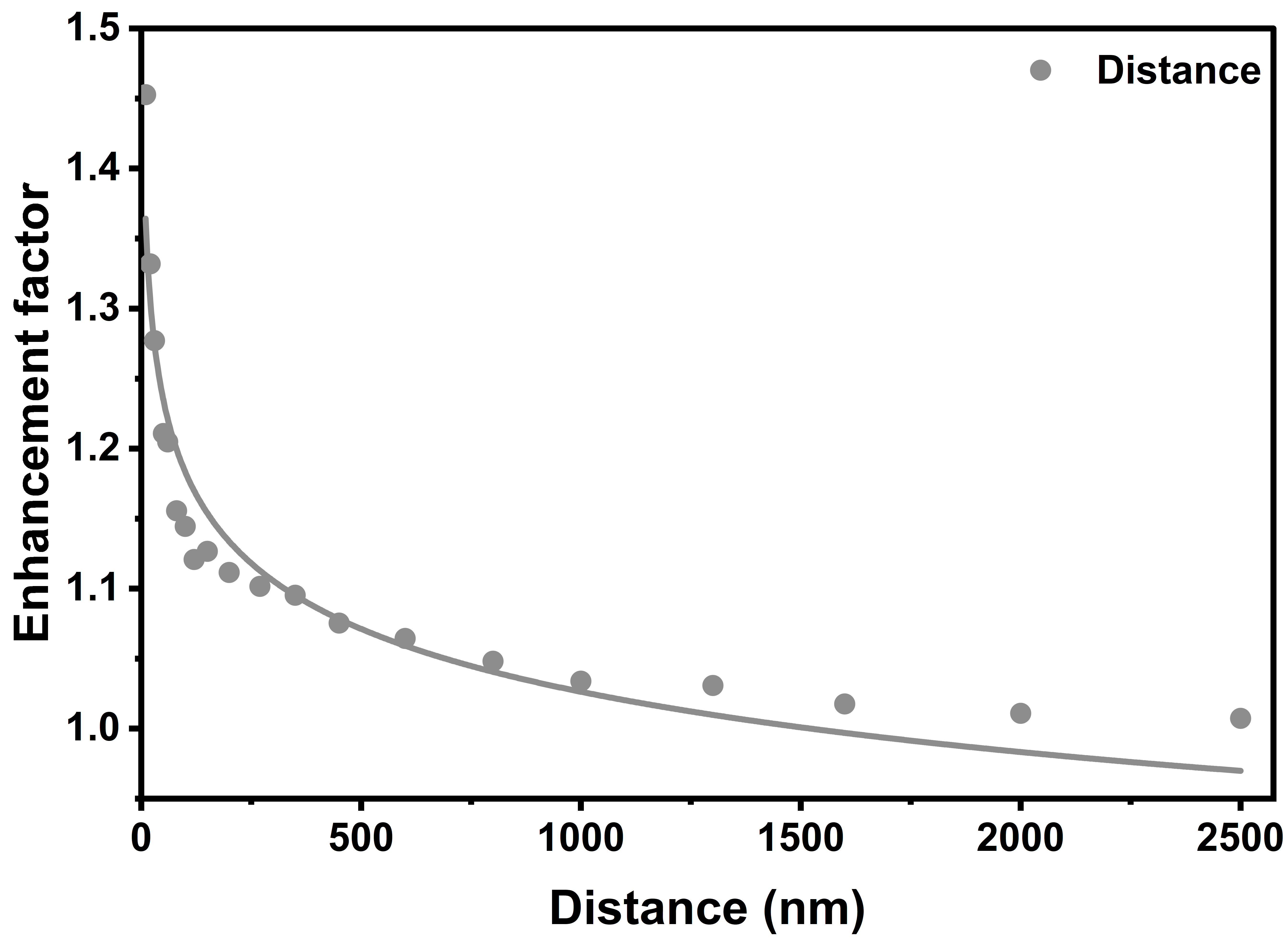


***Figure 1.*** Enhancement factor of the AuNS absorption cross-section at different distances between AuNS and ND from FDTD simulations. The enhancement factor of the AuNS absorption interface decreases with the increase in distance.


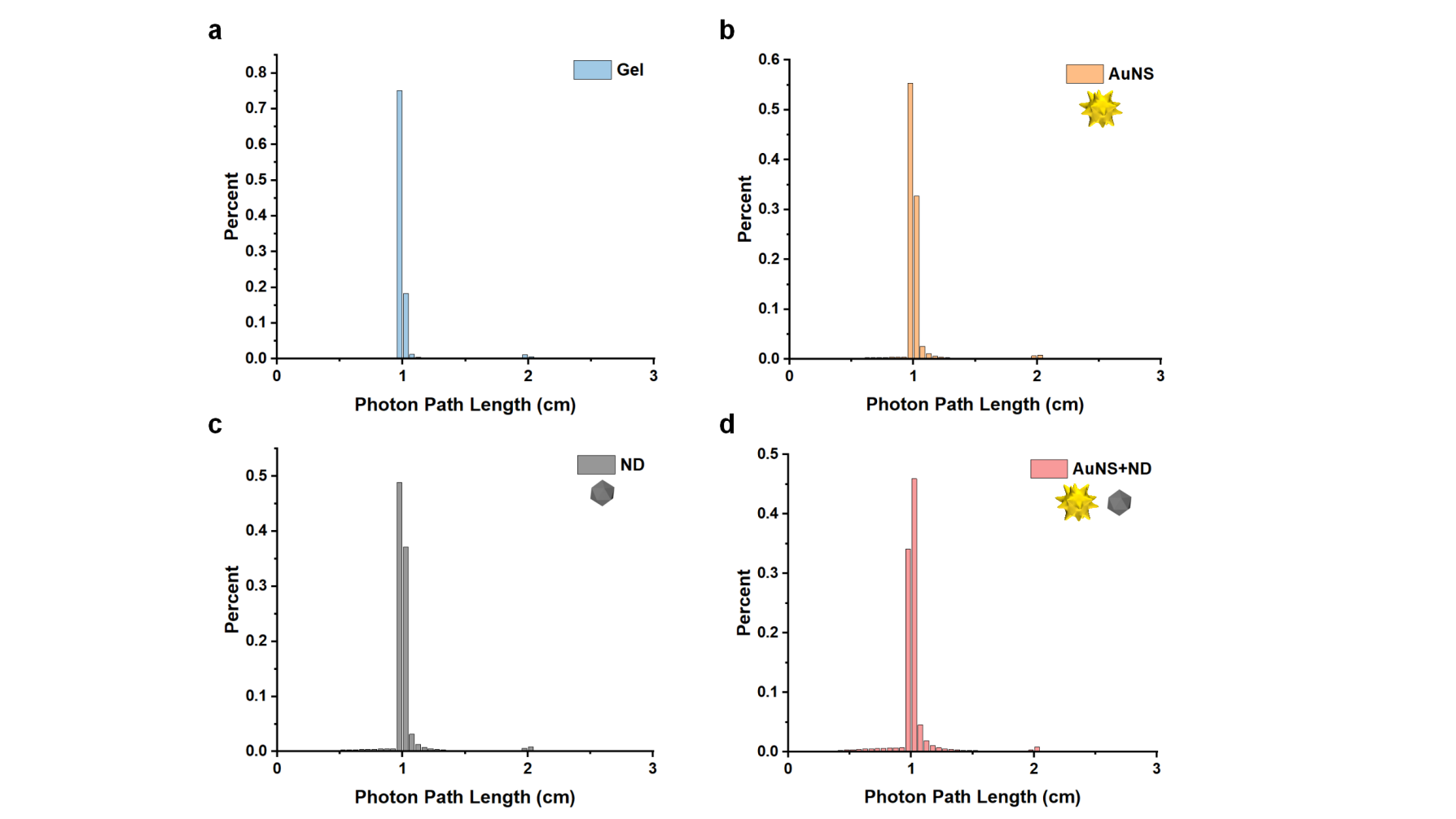


***Figure S2.*** Statistical graphs of photon path lengths in different systems. X-axis: Photon Path Length (mm); Y-axis: Percentage of photons with different path lengths relative to the total number of photons.


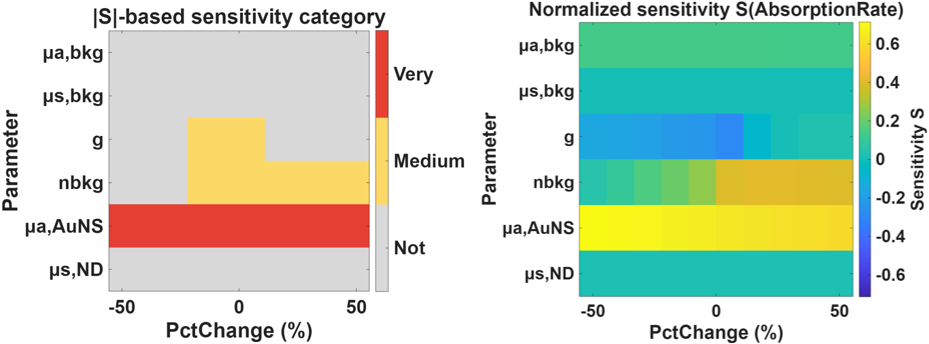


***Figure S3.*** Results of the sensitivity analysis for optical parameters based on Monte Carlo simulations. The left panel shows the sensitivity classification of different parameters according to the variation in global light absorption rate, which is divided into three categories: highly sensitive (red), moderately sensitive (yellow), and insensitive (grey). The global light absorption rate is highly sensitive to μ_a,AuNS_, moderately sensitive to g and n, and insensitive to μ_a,bkg_, μ_s,bkg_ and μ_s,ND_.

The right panel represents the normalized sensitivity of each optical parameter, where larger values indicate a greater influence on the absorption rate.


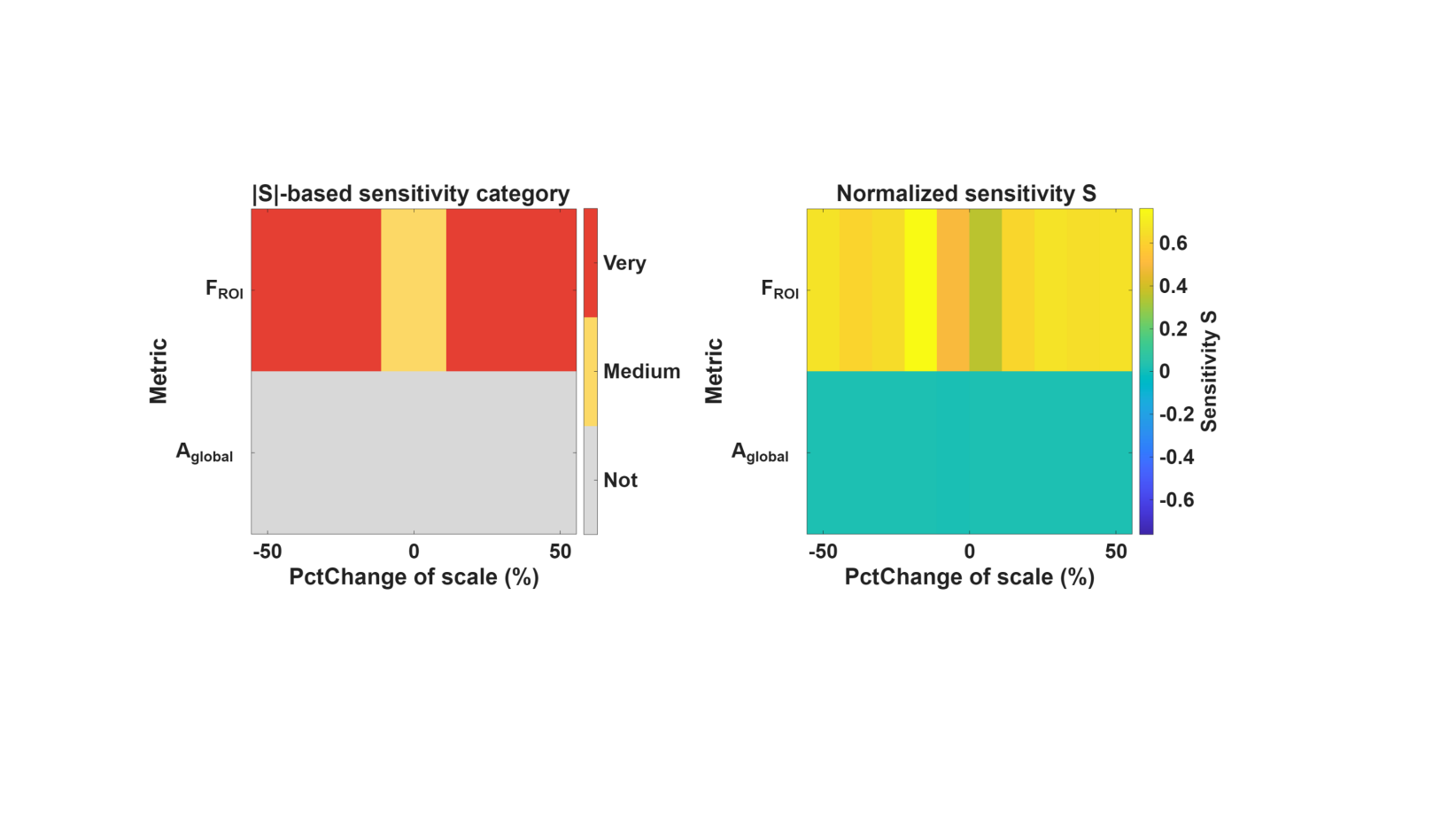


***Figure S4.*** Sensitivity analysis of the effect of variations in the scattering coefficient μ_s,ND_ on the spatial distribution of energy deposition (i.e., spatial light distribution). (Left) Sensitivity classification based on the magnitude of sensitivity (|S|). The region of interest (F_ROI_) exhibits very high sensitivity (red region), while the global light absorption region (A_global_) shows negligible variation. The results demonstrate that F_ROI_ is highly sensitive to changes in the scattering coefficient, indicating that such variations exert a crucial influence on light distribution.

(Right) Normalized sensitivity S as a function of the scattering coefficient, where larger values correspond to a greater influence.


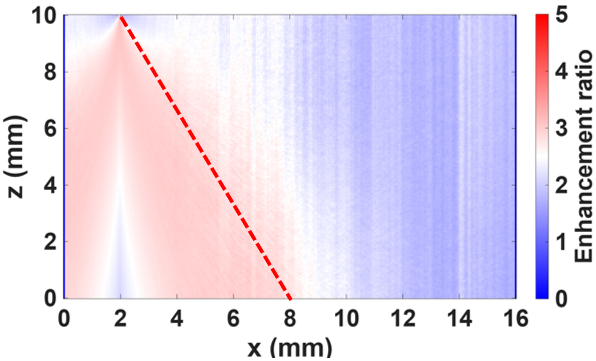


***Figure S5.*** Schematic diagram of the statistics of near-infrared photon density and near-infrared absorption at different positions from the light source. The white dashed line indicates the position to be counted.

**Materials and Reagents**

ND with individual sizes of 10 nm, which are synthesized by detonation techniques, were supplied by Gansu Gold Stone Nano. Material. Co. Ltd. (Gansu, China). Fluorescent ND (1 nM) were purchased from Adamas Nanotechnologies (USA). PDDA, AgNO3, HAuCl4 and AA were purchased from Aladdin Biochemical Technology (China). DMEM Medium with High Glucose, Phenol Red-free DMEM Medium, Phosphate-Buffered Saline (PBS), Agarose (ST004L), Lyso-Tracker Green (C1047S) and Calcein-AM (C1371S) were purchased from [Beyotime Biotechnology](https://www.beyotime.com/index.htm) (China). Cell Culture Flask (T25), Culture Dish (Diameter 35 mm) were purchased from Tansoole (China). Thermocouple (TT-K-30), thermocouple thermometer (YET-640) were purchased from Kepco (China). Shaking Incubator (NY-H1S) was purchased from Enyitools (China). S. aureus (ATCC43300) was purchased from Shifeng biological technology (China). LB Agar and LB Broth Powder were purchased from Coolaber (China). UV-vis spectrophotometer (UV-1900i) was purchased from Shimadzu (Japan). An 808 nm near-infrared laser (RAL808T1-8W) was purchased from Beijing Rayyuan Technology (China). Ultrapure water (18.2 MΩ cm at 25 ℃) produced by a Millipore purification system (Milli-Q IQ Element, Merck, USA) was used throughout our experiments. Adult female BALB/c nude mice (6-8 weeks old) were purchased from the Animal Center of Wuhan University. Human B-cell lymphoma Ramos cells were purchased from Cell Bank of the Chinese Academy of Sciences (CAS).

**S5 Preparation of Nanoparticle/Agarose gel Models**

**2% Agarose Gel:** Dissolve 0.4 g of agarose powder in 20 ml of deionized water. Heat the mixture to boiling and remove it from the heat immediately, shake it thoroughly, then reheat to boiling. Repeat this process three times to obtain a homogeneous and transparent solution. Subsequently, transfer the transparent solution into a 12-well or 24-well plate and allow it to cool and solidify at room temperature (30 min), forming cylindrical agarose gels.

**ND/Agarose Gel (3.2×10⁻³ v%):** Dissolve 0.4 g of agarose powder in 20 ml of deionized water, then add 224 μl of 10 mg/ml ND suspension to the mixture and shake well. Heat the mixture containing the ND suspension to boiling, remove it from the heat immediately, shake it thoroughly, and reheat to boiling. Repeat this step three times to achieve a uniformly mixed solution (the solution appears turbid due to the presence of ND). Next, transfer the ND-containing agarose solution into a 12-well or 24-well plate and let it cool and solidify at room temperature (30 min), yielding cylindrical ND/agarose gels.

**AuNS/Agarose Gel (3.6 pM):** Dissolve 0.4 g of agarose powder in 20 ml of deionized water. Heat the mixture to boiling and remove it from the heat immediately, shake it thoroughly, then reheat to boiling. Repeat this process three times to obtain a homogeneous and transparent solution. Then, 1000 μL of the above agarose solution was transferred to well plates, and 100 μL of 39 pM AuNS solution was added. The mixture was thoroughly mixed by pipetting and stirring, then cooled and solidified at room temperature (30 min) to form cylindrical AuNS/agarose gels.

**AuNS+ND/Agarose Gel (ND: 3.2×10⁻³ v%, AuNS: 3.6 pM):** Dissolve 0.4 g of agarose powder in 20 ml of deionized water, then add 224 μl of 10 mg/ml ND suspension to the mixture and shake well. Heat the ND-containing mixture to boiling, remove it from the heat immediately, shake thoroughly, and reheat to boiling; repeat this cycle three times to obtain a homogeneous solution. Then transfer the ND-containing agarose solution into a 12-well or 24-well plate. Dispense 1000 μl of the ND-agarose solution into each well, followed by adding 100 μl of 39 pM AuNS solution. Mix the solution thoroughly by pipetting up and down, and allow it to cool and solidify at room temperature to form cylindrical ND+AuNS/agarose gels.


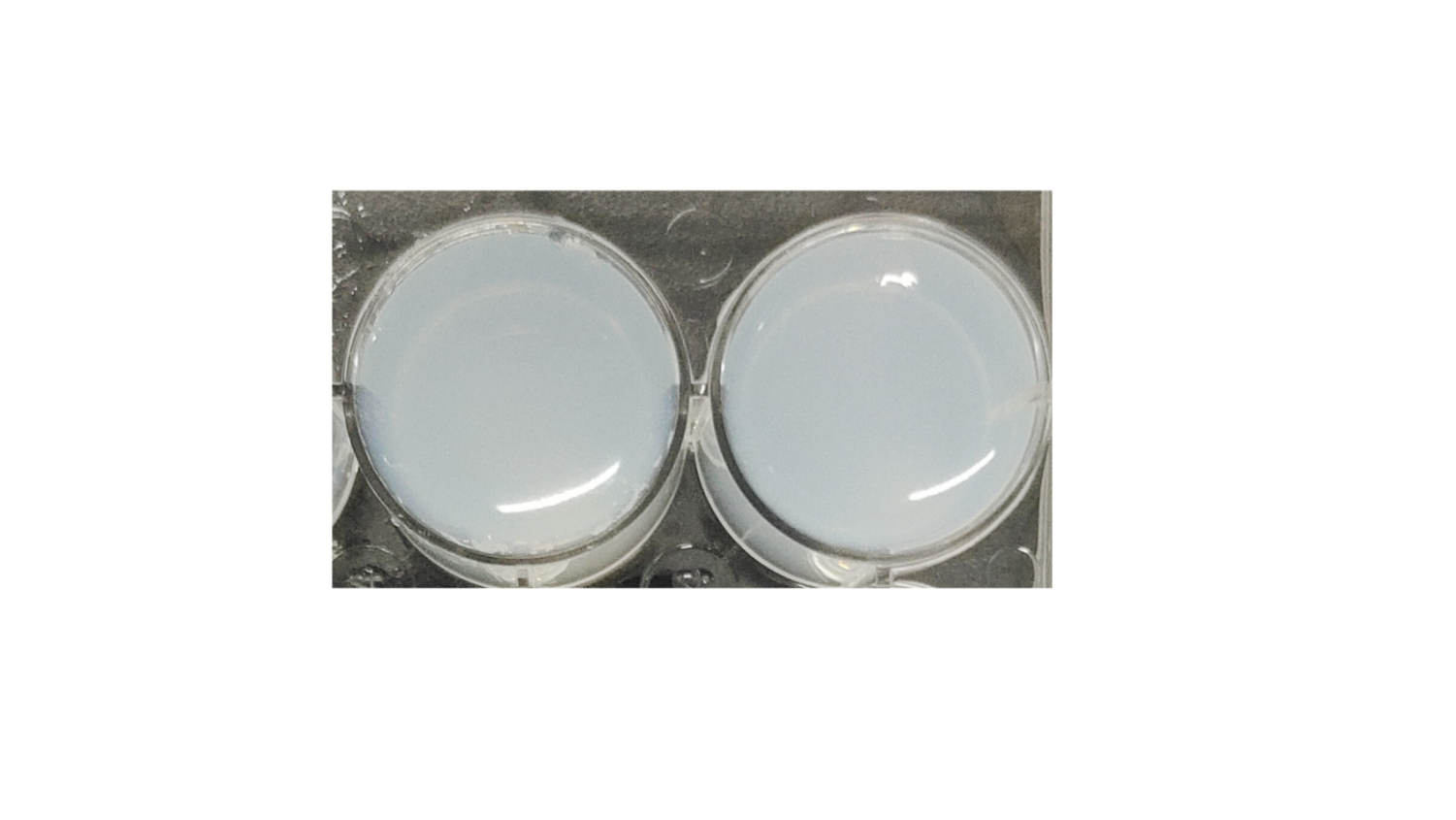


***Figure S6.*** Self - made agarose gel model in a 12 - well plate.

**S6 Synthesis of Gold Nanostars**

The synthesis of gold nanostars was achieved through a gold seed-mediated reduction method^3^. Initially, 3000 mg of poly (diallyl dimethyl ammonium chloride) (PDDA) was added to 30 mL of deionized water, followed by the sequential addition of 1500 μL of 5 mM hydrogen aurate (HAuCl4), 300 μL of 8 mM silver nitrate (AgNO3), 300 μL of 100 mM ascorbic acid (AA), and 300 μL of gold seeds. The mixture was stirred overnight at room temperature, after which the product was collected the next day and concentrated by centrifugation at 10000 rpm revolutions per minute.

**S7 Transmission Electron Microscopy Imaging of Nanoparticles**

Both ND and AuNS were characterized by taking images using a transmission electron microscope F200 (JEOL Ltd., JEM - F200).


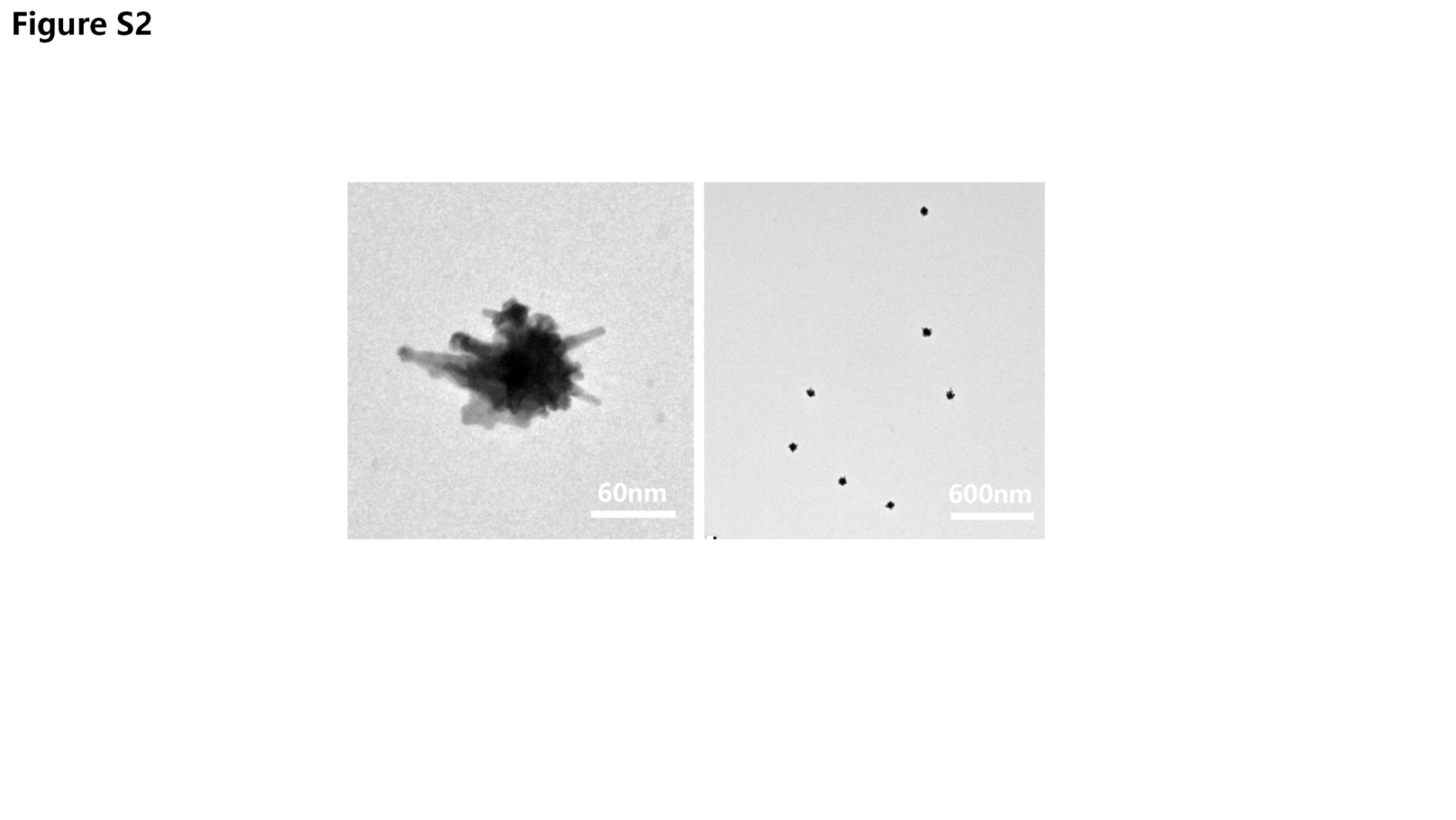


***Figure S7.*** TEM of AuNS


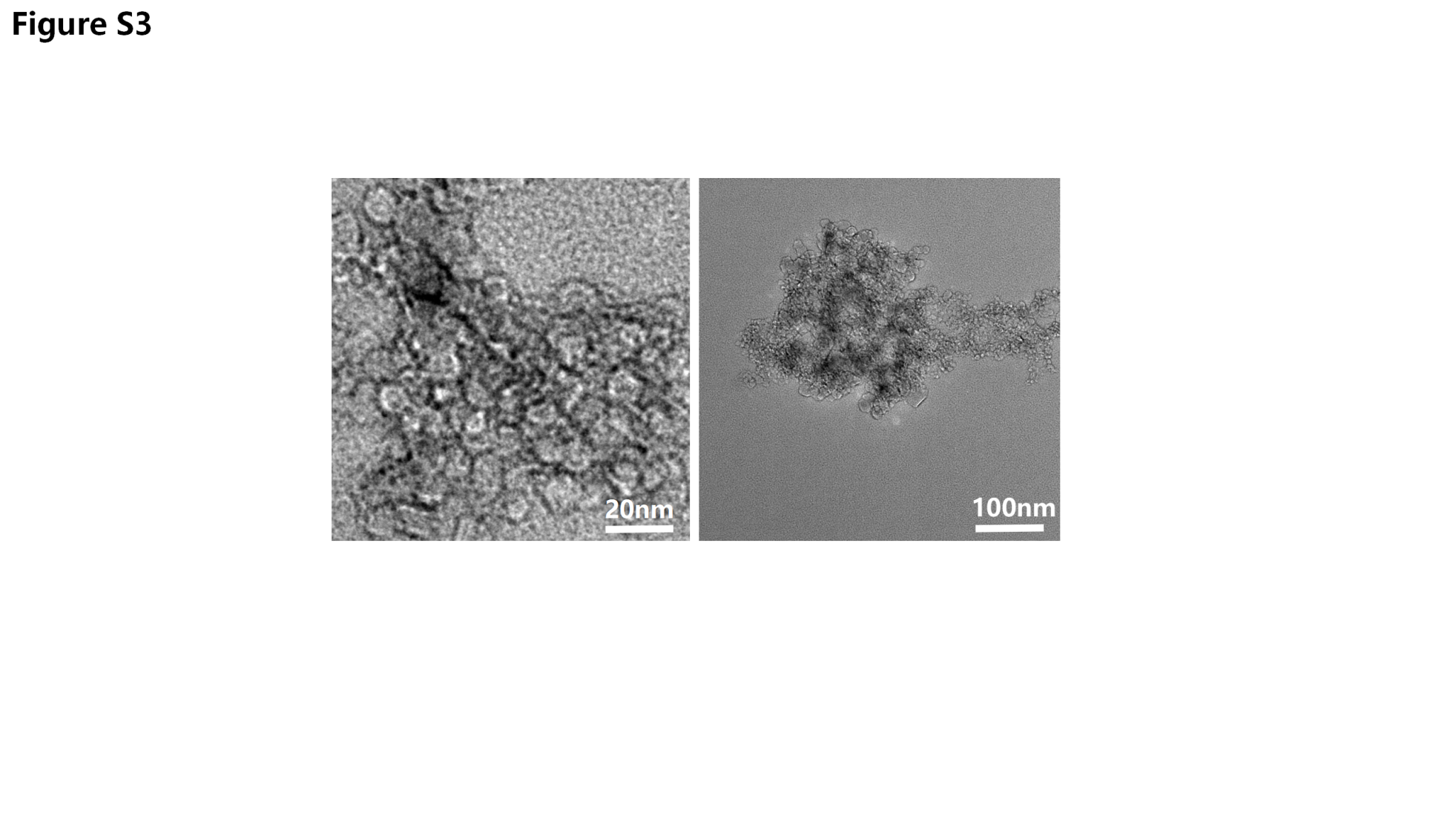


***Figure S8.*** TEM of ND

**S8 Experimental Set-up**

A 500 mW, 808 nm near - infrared laser (RAL808T1 - 8W) was used as a heat source, aiming at a vertically placed cylindrical agarose sample. In a range of 12 mm, a thermocouple with a 1-mm tip size was placed every 3 mm, and these thermocouples were embedded in the gel. A 4 - channel digital thermocouple thermometer was used to record the temperature once every minute for 20 minutes. To maintain thermal insulation, the agarose hydrogel was placed on an insulated plastic petri dish.


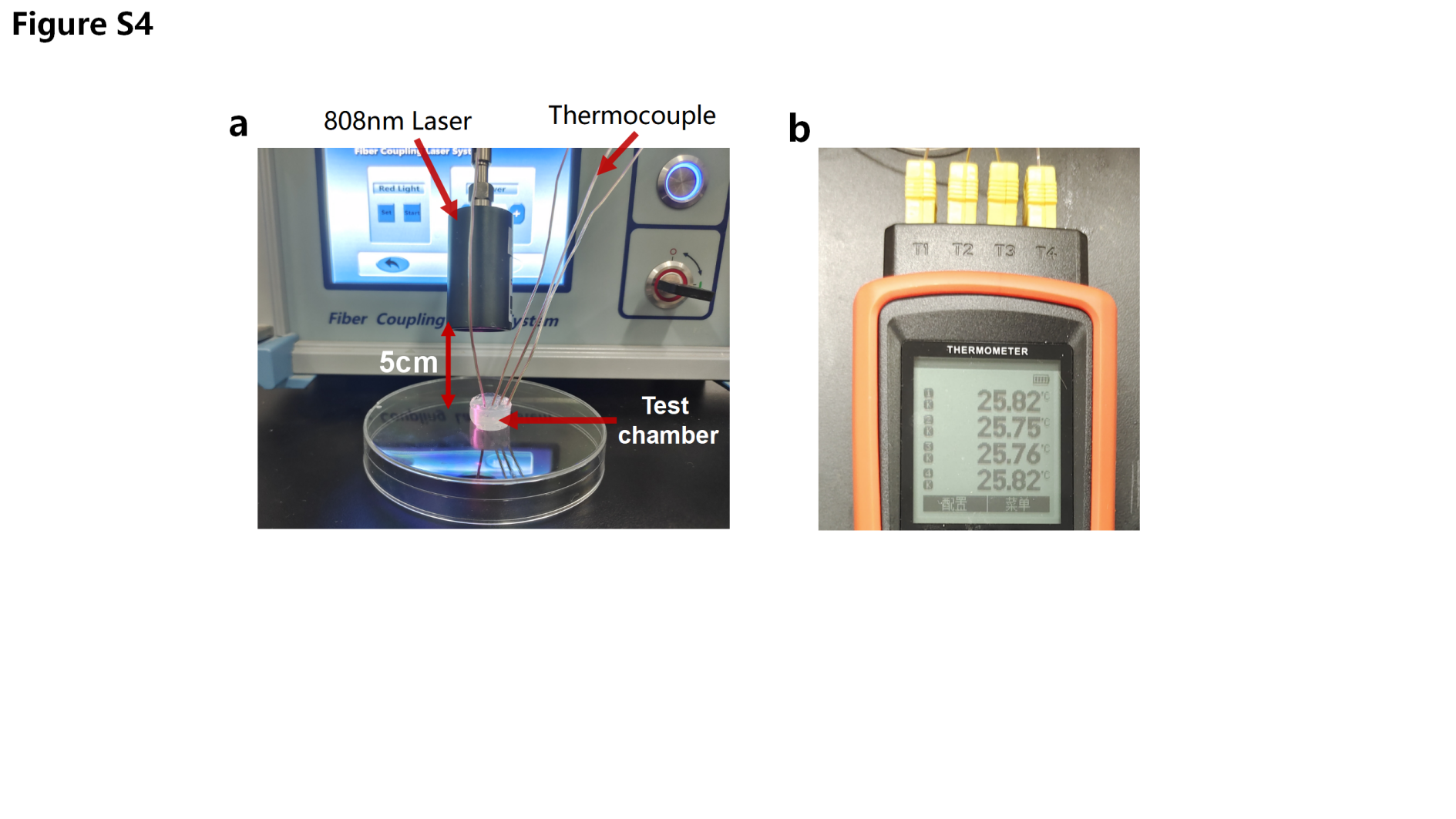


***Figure S9.* (a-b)** Photographs for the experimental set-up. (**b)** A digital thermocouple thermometer

**S9 Heat Transfer Profiles of ND**

To study the heat flow properties of the ND - particle gel, heat transfer measurements were carried out. Cylindrical agarose gels were used as the basal medium. During the preparation process, different volumes of ND solution were added to the agarose, followed by vigorous stirring at high temperature to uniformly disperse the ND particles in the agarose. The agarose gels were prepared using a 12 - well plate, and then the temperature distribution of the ND - agarose gels with three concentrations was measured using a thermocouple and a digital thermocouple thermometer.


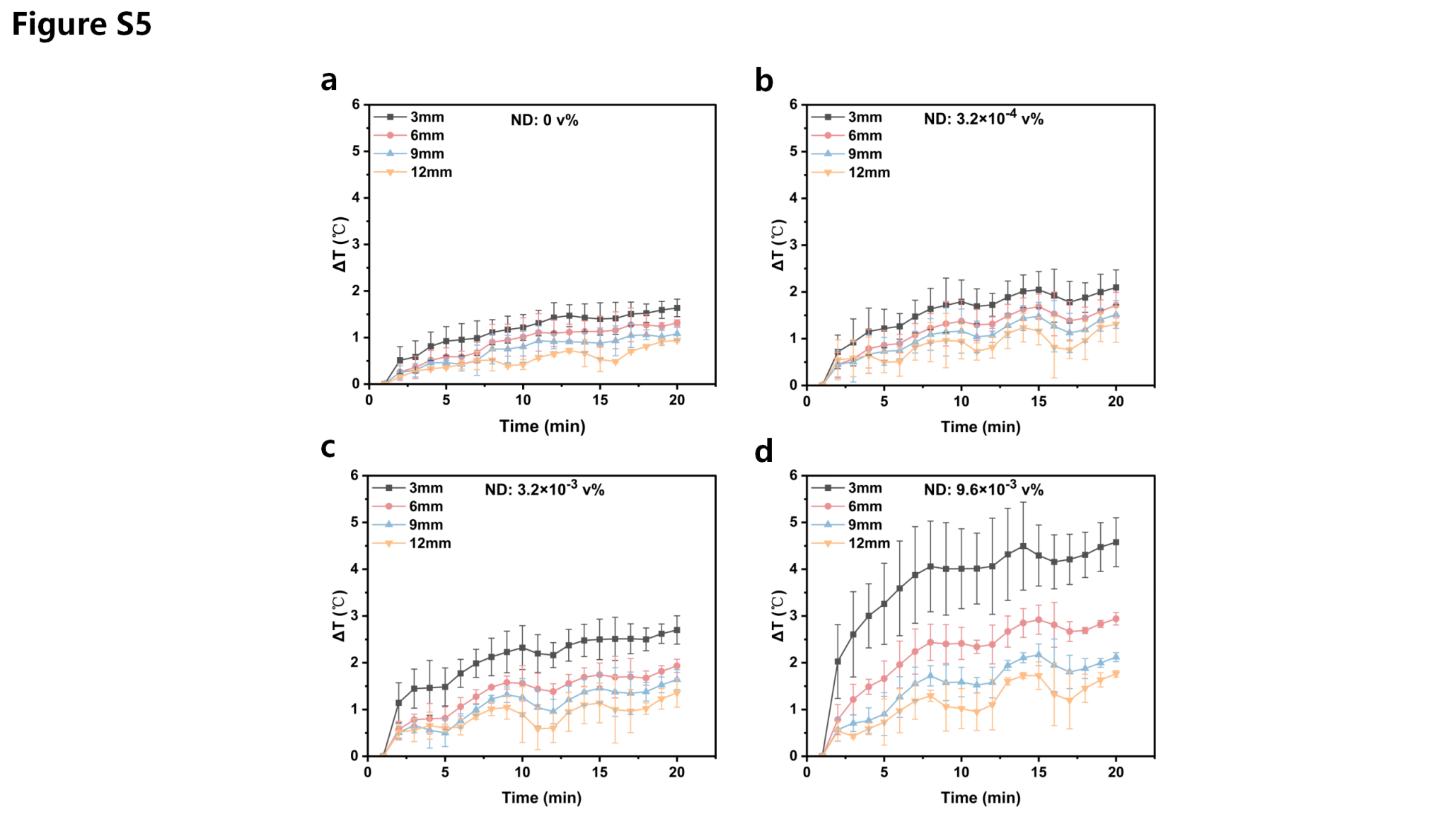


***Figure S10.*** Temporal heat transfer profiles of different volume percentages of ND in 2% agarose gel base medium. **(a)** 0 v%, **(b)** 3.2 x 10^-4^ v%, **(c)** 3.2 x 10^-3^ v%, **(d)** 9.6 x 10^-3^ v%, (n=3). Symbols represent the mean value and vertical bars indicate the standard deviation.

**S10 Heat Transfer Profiles of ND Gel as a Function of Distance Form the Heat Source**

The relationship between the heat transfer curve of the ND gel and the distance from the laser heating source is shown in Figure S8. The first thermocouple is closest to the laser heating point, so the temperature change is the most significant. The thermocouple farthest from the laser heating point has the smallest temperature mperature differencechange. The te (ΔT) decreases as the distance from the heat source increases. This can be explained by the heat loss to the surrounding environment, which reduces the amount of heat transferred to more distant locations.


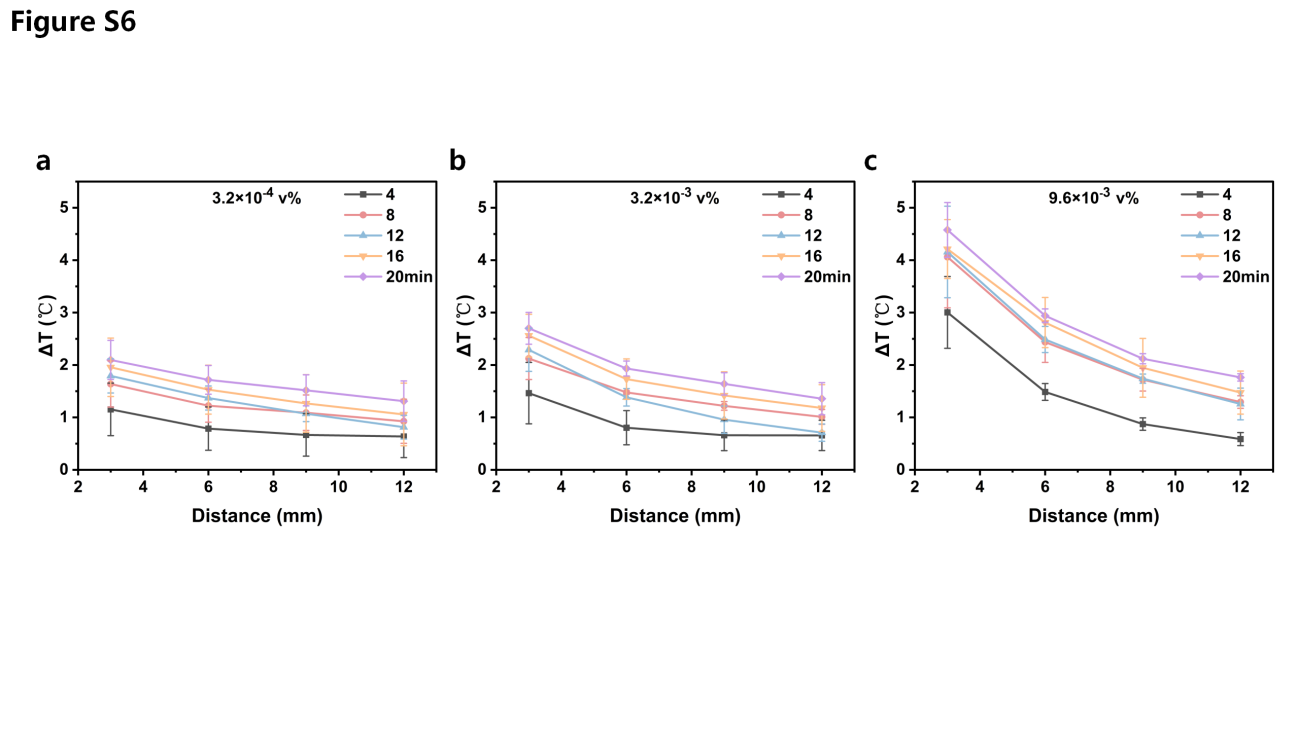


***Figure S11.*** Heat transfer profiles of ND Gel as a function of distance of three different volume percentages of ND in 2% agarose. **(a)** 3.2 x 10^-4^ v%, **(b)** 3.2 x 10^-3^ v%, **(c)** 9.6 x 10^-3^ v%, (n=3). Symbols represent the mean value and vertical bars indicate the standard deviation.

**S11 Extinction Measurement**

The extinction was measured using a UV-vis spectrophotometer (Shimadzu, UV-1900i). Each sample was scanned at a scanning rate of 700 nm per minute. The wavelength range of light was from 400 to 1100 nm. The extinction at 808 nm was of particular concern because the wavelength of the laser beam used for heating the samples was the same as that of the light source in the UV-vis spectrophotometer.

Measurements of the extinction of ND gels and ND suspensions were carried out. It was found that, at the same concentration, the extinction of the ND gel was significantly higher than that of the ND suspension. It can be assumed that there is some mechanism within ND itself that increases the extinction of the ND gel and enhances photothermal heating.


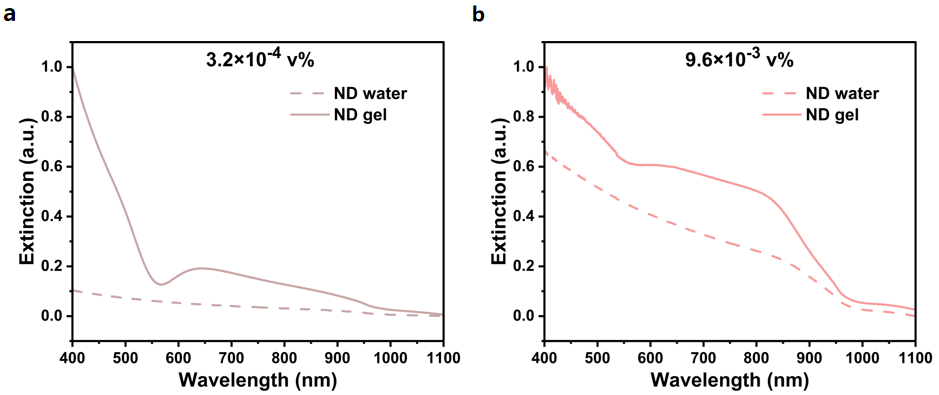


***Figure S12.*** Extinction spectra of ND suspensions in water and ND gels. **(a)** 3.2 x 10^-4^ v %, **(b)** 9.6 x 10^-3^ v %.

**S12 Integrating-Sphere Measurement**

The reflectivity of nanodiamond (ND) was measured using the integrating sphere mode of a UV-visible spectrophotometer to verify light scattering as the dominant optical property of ND. The ND powder was directly used as the test sample. The experimental parameters were set as follows: a scanning rate of 700 nm/min, a wavelength range of 400–1100 nm, and three replicate measurements.

The experimental results showed that the ND powder exhibited an extremely high reflectivity, reaching up to 93.7% at 808 nm. Therefore, it can be concluded that the dominant optical property of ND is light scattering, while its light absorption capacity is negligible.


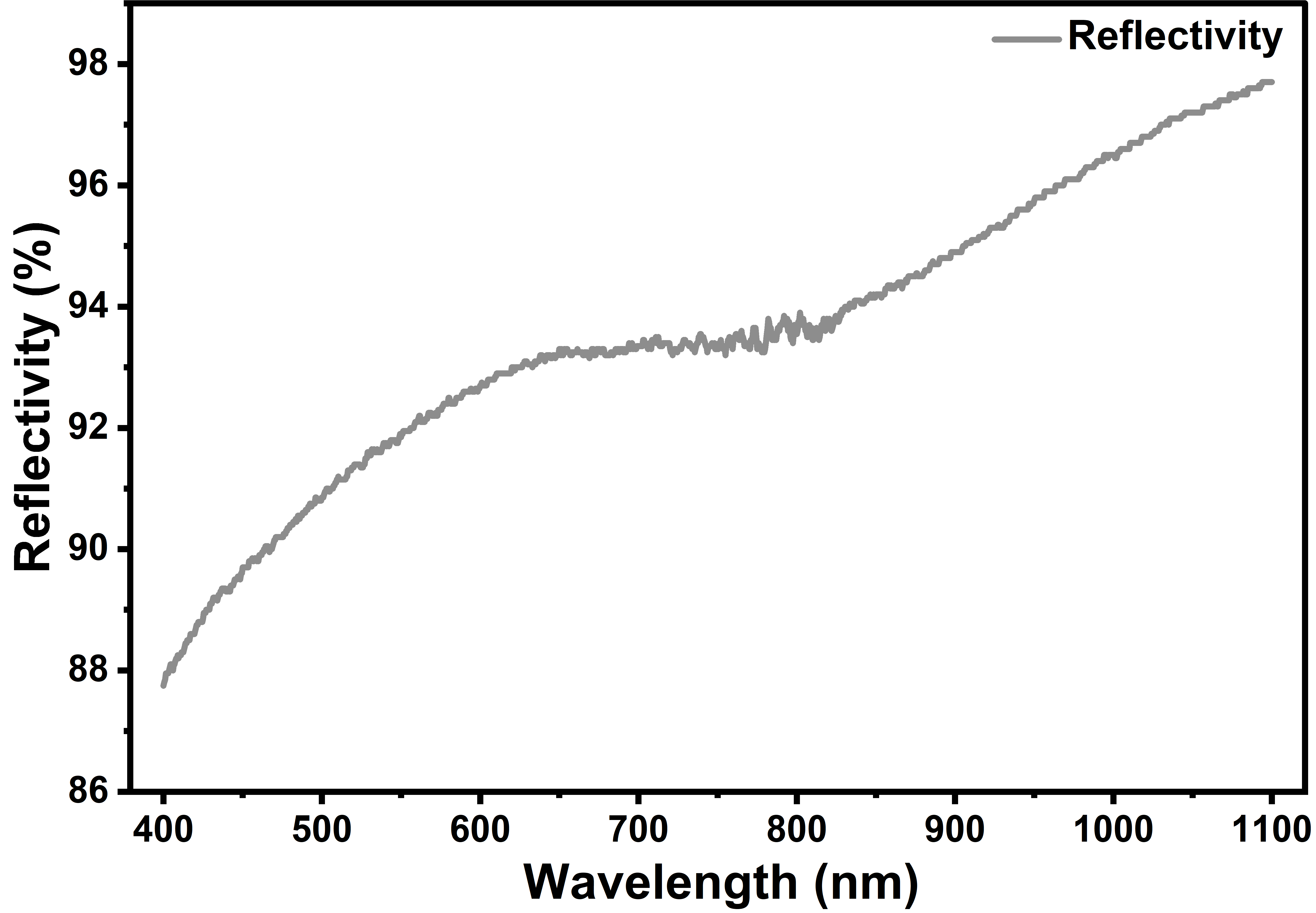


***Figure S13.*** The reflectivity of ND powder measured in the integrating sphere mode.

**S13 Heat Transfer Profiles of ND-AuNS Gel as a Function of Distance Form the Heat Source**

The relationship between the heat transfer curve of the ND - AuNS gel and the distance from the laser heating source is shown in Figure S10. Compared with the ND gel, after adding AuNS, the temperature change at each distance increases, and photothermal heating is enhanced. However, its characteristics are similar to those of the ND gel, with the temperature difference (ΔT) decreasing as the distance from the heat source increases.

**
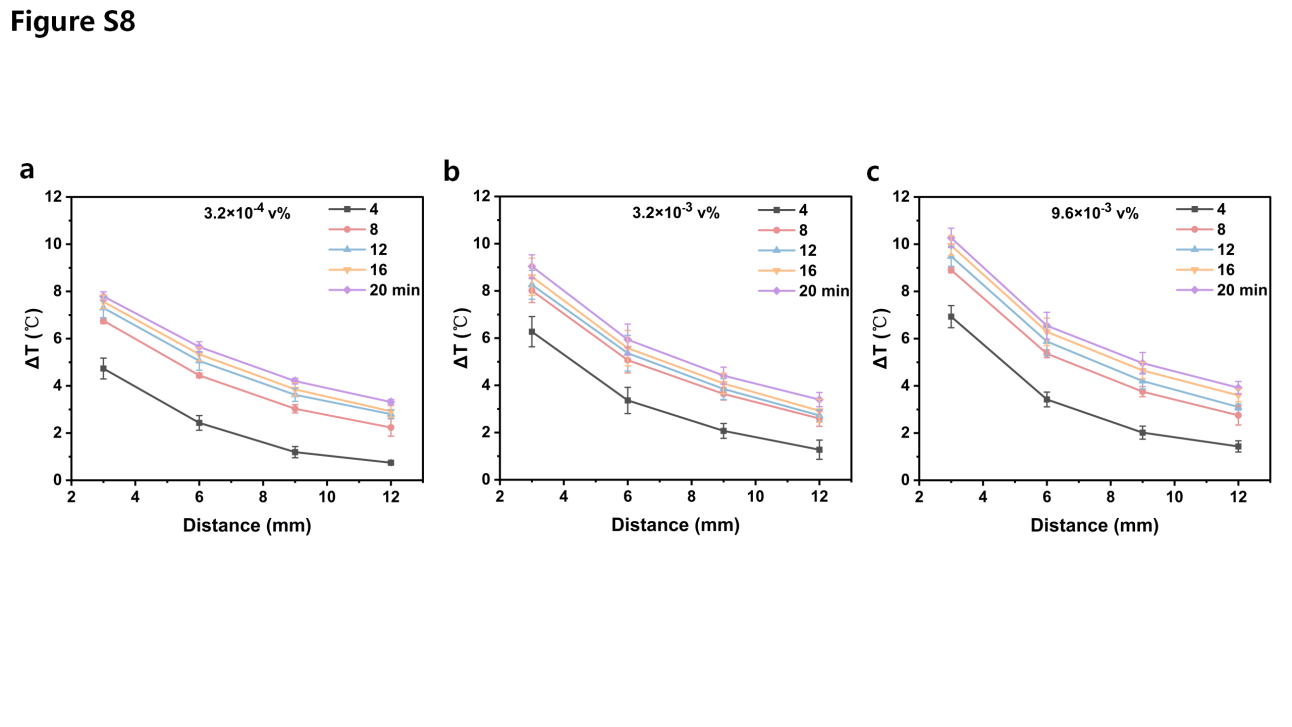
**

***Figure S14.*** Heat transfer profiles of ND-AuNS gel as a function of distance of three different volume percentages of ND in 2% agarose. **(a)** 3.2 x 10^-4^ v%, **(b)** 3.2 x 10^-3^ v%, **(c)** 9.6 x 10^-3^ v%, (n=3). Symbols represent the mean value and vertical bars indicate the standard deviation.

**S14 Extinction Measurement of AuNS+ND Agarose gel**

The change in extinction of AuNS gel after the addition of ND was also measured using a UV-visible spectrophotometer. Each sample was scanned at a scanning rate of 700 nm per minute. The wavelength range of light was from 400 to 1100 nm. Three groups of ND concentrations were set, namely 3.2×10⁻⁴, 3.2×10⁻³, and 9.6×10⁻³ v%. After obtaining the extinction spectra, the extinction values at 808 nm for different groups were collated and compared.

The results of the column statistical analysis showed that the absorbance of AuNS hydrogels increased significantly after the incorporation of ND, and the extinction values rose with the increase in ND concentration. These findings indicate that the light-scattering capability of ND can significantly enhance the light absorption capacity of AuNS, demonstrating that the light-harvesting efficiency of the composite system is improved.


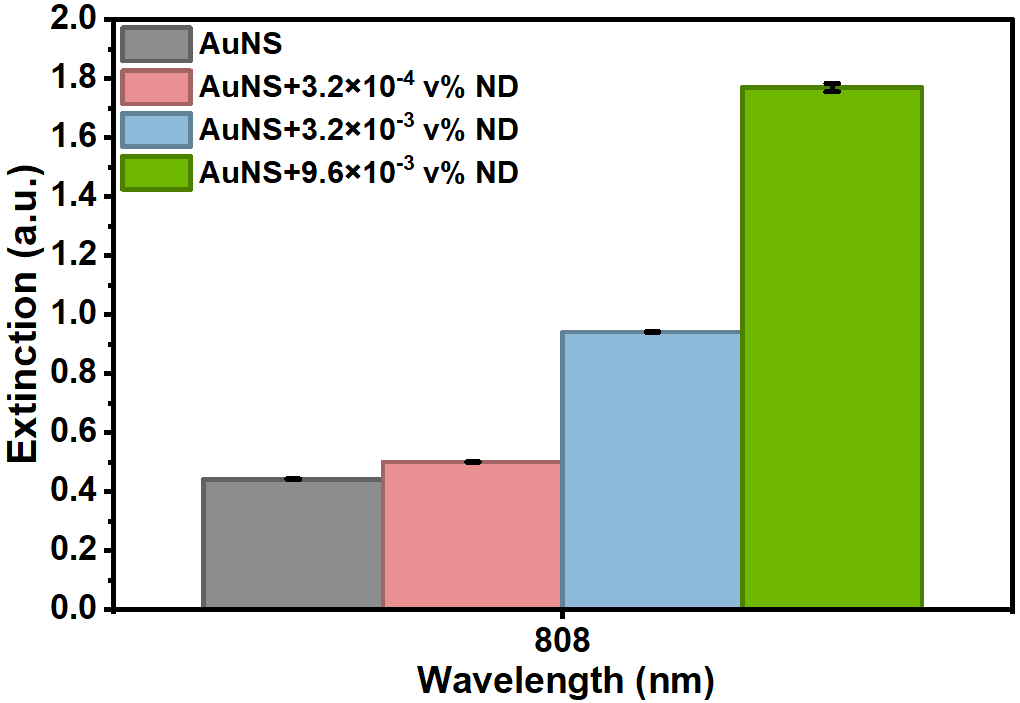


***Figure S15.*** Statistical analysis of extinction of AuNS+ND composite gel at 808 nm (n=3).

**S15 Photothermal sterilization experimental**

First, add nanoparticles to a petri dish (with a diameter of 35 mm), then add LB medium that has been autoclaved under high pressure steam. Shake well to evenly distribute the nanoparticles in the LB medium (the final concentration of AuNS is 3.6 pM, and the final concentration of ND is 3×10⁻³ v%). After a period of time, when the medium solidifies, spread Staphylococcus aureus (Shifeng, ATCC43300) on the medium using the spread - plate method. Incubate in a constant - temperature incubator at 37 °C for 2 h, then irradiate the middle position of the medium for 15 min with a near - infrared laser (500 mW, at a height of 10 cm). After the irradiation, place it back in the 37 °C constant - temperature incubator and incubate overnight. The next day, count the number of colonies in the irradiated area and conduct a difference analysis.


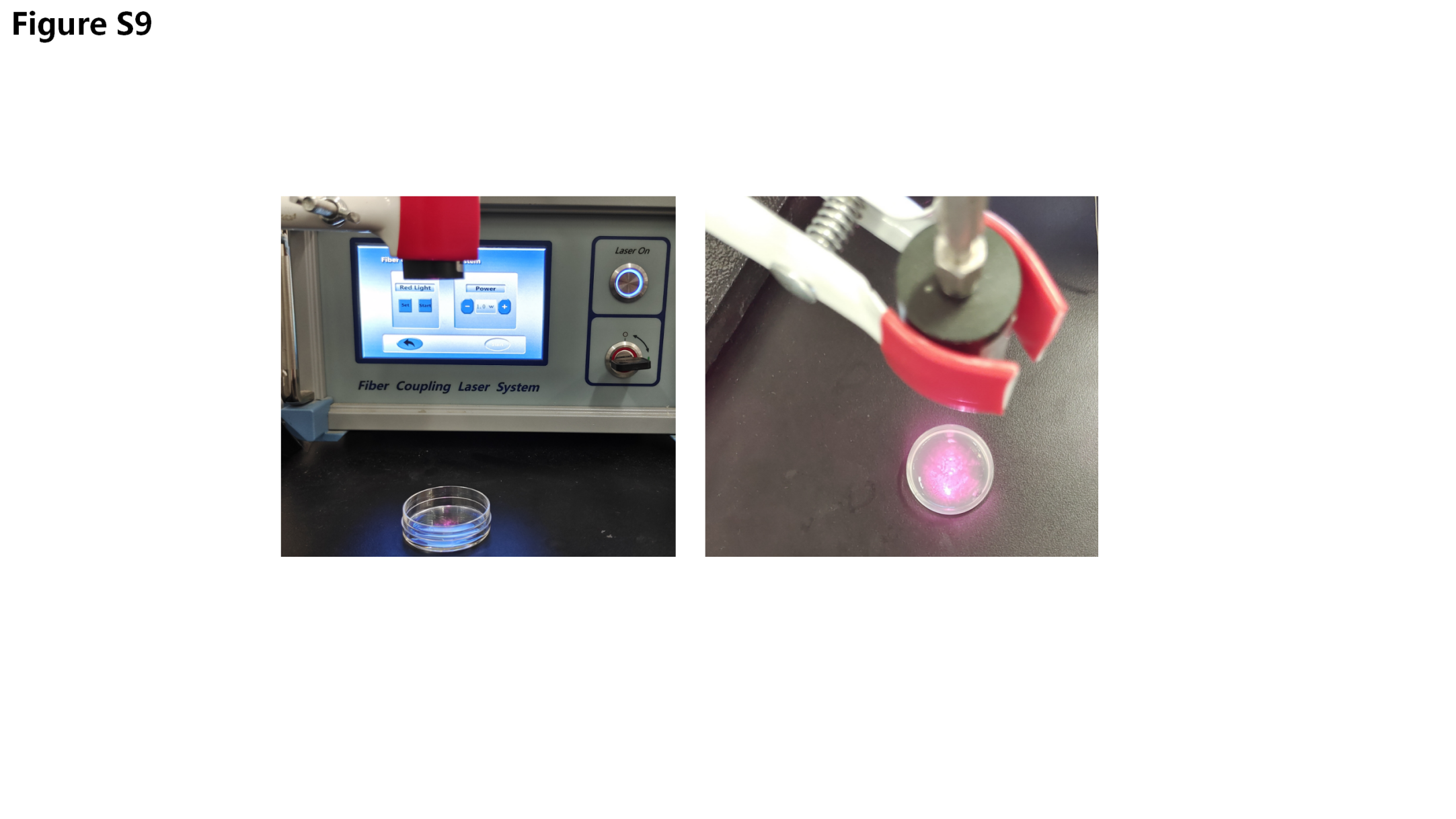


***Figure S16.*** Photographs of the photothermal sterilization experimental device.


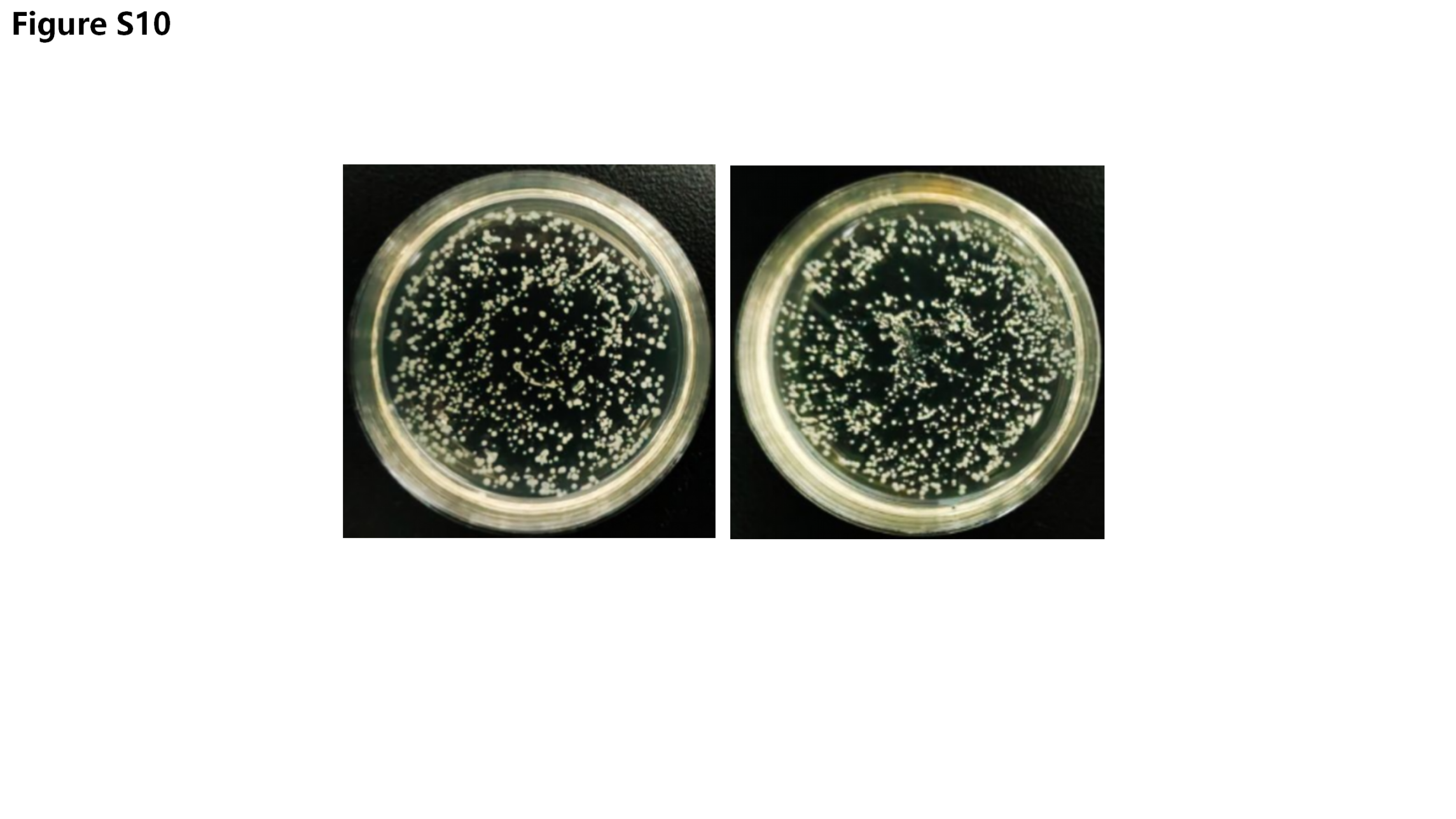


***Figure S17.*** Photographs of bacterial experiments (without NIR).

**S16 Cell dark - field and fluorescence imaging**

Plant Hela cells at an appropriate density in a petri dish (with a diameter of 60 mm) (60 mm culture dish was used to meet the requirements of dark-field imaging), add 2 ml of DMEM medium. Subsequently, add nanoparticles to the medium (the final concentration of AuNS is 1.8 pM, and the final concentration of ND is 7.5×10⁻⁴ v%). Shake well to ensure the nanoparticles are evenly distributed in the DMEM medium. Place it in a carbon dioxide incubator and culture overnight to allow the cells to fully endocytose the nanoparticles. The next day, when the cells grow to an appropriate density, under a microscope, use a condenser in dark - field mode and take pictures with a color CCD; In TIRF mode, using a 532 nm laser for excitation, observe the distribution of fluorescence within the cells.

**Optical Measurement Setup：**

Optical measurements were carried out using a customized wide-field imaging and in situ single-particle spectroscopy system under room temperature conditions.

Wide-field fluorescence imaging: a 532 nm laser (OBIS laser box, Coherent, Inc., USA) was transmitted through a 532 ± 2 nm band-pass filter and a 532 nm long-pass dichroic mirror and then focused on the sample using a 40× objective lens (NA = 0.60). The photoluminescence (PL) of the sample was separated from the 532 nm laser by the above-mentioned dichroic mirror. Subsequently, the PL passed through a 610 nm long-pass filter and reached a back-illuminated scientific CMOS (KURO 1200B, Princeton Instruments, USA) for wide-angle fluorescence imaging.

In situ single-particle fluorescence spectroscopy: A 532 nm laser (OBIS laser box, Coherent, Inc., USA) was transmitted through a 532 ± 2 nm band-pass filter and a 532 nm long-pass dichroic mirror and then focused on the sample using a 40× objective lens (NA = 0.60). The photoluminescence (PL) of the sample was separated from the 532 nm laser by the above-mentioned dichroic mirror and then passed through a 537 nm long-pass filter. The region of interest (ROI) was manually defined, and the PL within this ROI was directed to a grating spectrometer (SpectraPro HRS-500, Princeton Instruments, Inc., USA) equipped with a 600 g/mm grating and a back-illuminated scientific CMOS (KURO 1200B, Princeton Instruments, USA) for in situ single-particle spectral measurements.

Wide-field scattering imaging and in situ single-particle scattering spectroscopy: An inverted microscope (Nikon, Japan) equipped with a dark-field condenser (numerical aperture (NA = 1)) and a 40× air objective lens (NA = 0.60) was used.


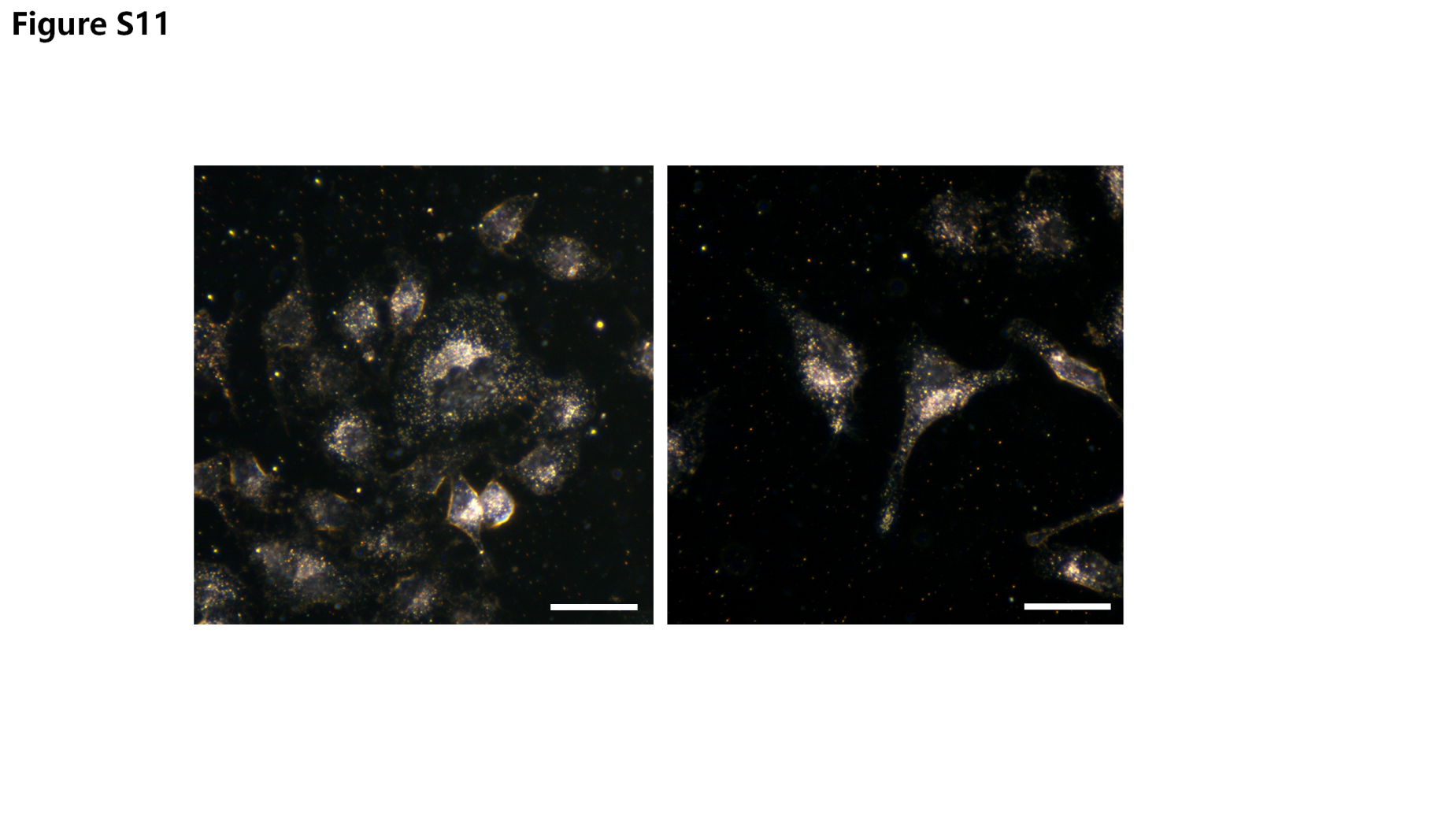


***Figure S18.*** Dark - field imaging of cells containing AuNS.


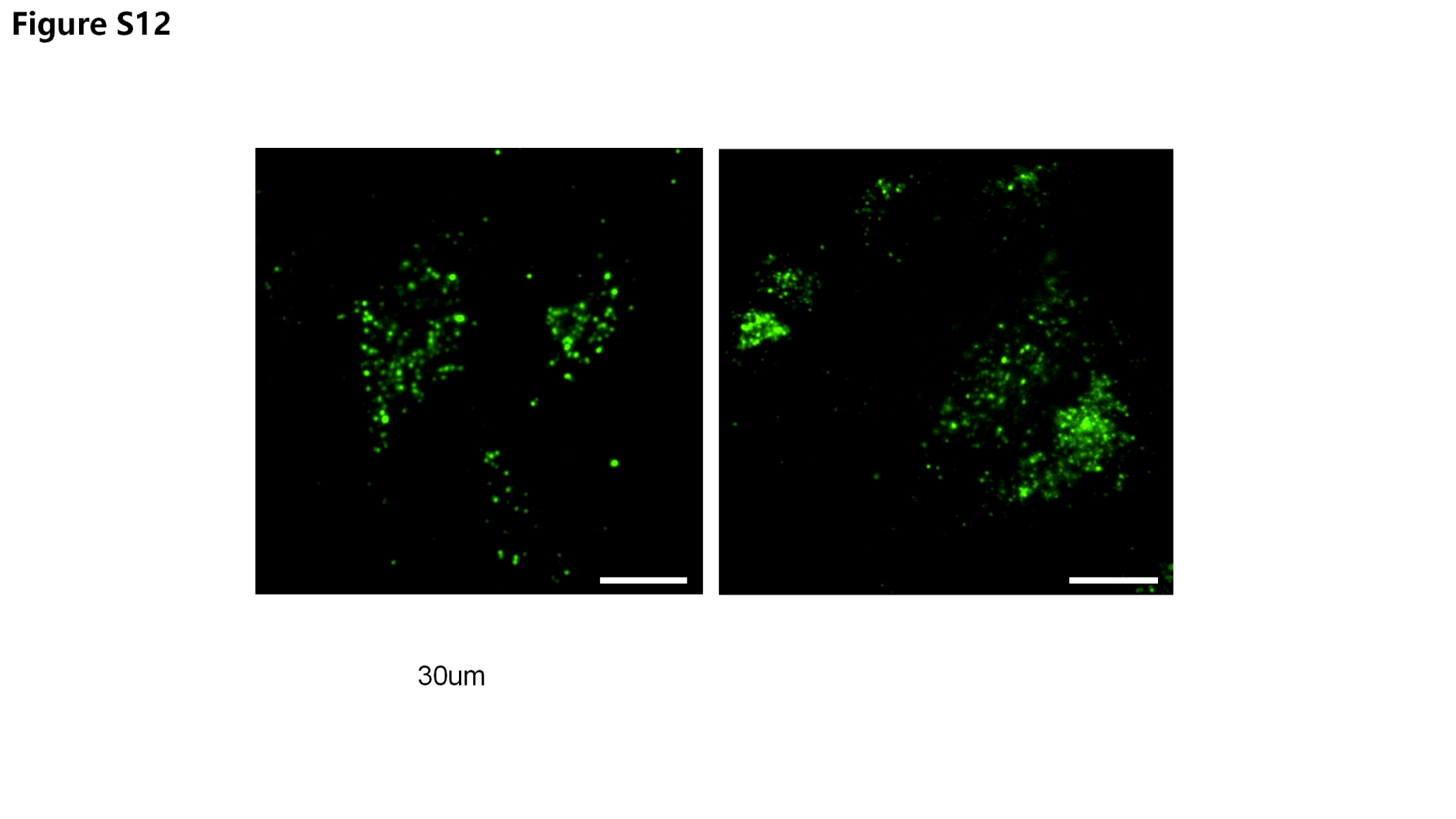


***Figure S19.*** Fluorescence imaging of cells containing ND. Scale bars: 30μm.

**S17 Dark - field scattering spectrum**

First, perform dark - field imaging using an inverted microscope equipped with a dark - field condenser (numerical aperture (NA = 1)) and a 40× air objective (NA = 0.60). Subsequently, select multiple relatively bright scattering spots within the cells for scattering spectrum acquisition (number of acquisitions ≥ 20). Integrate the collected data using Origin software to create a scattering spectrum diagram with error bars.


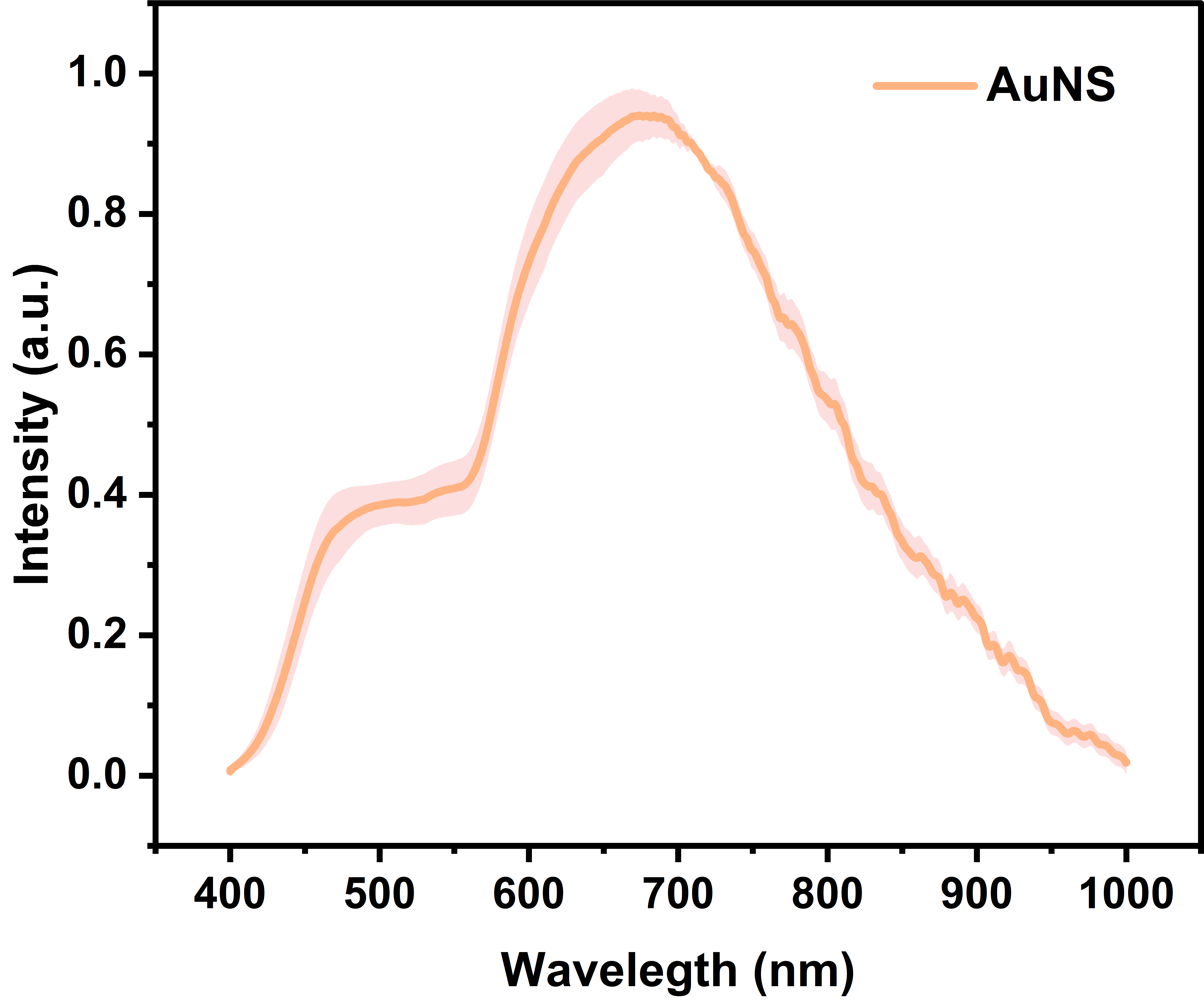


***Figure S20.*** A scattering spectrum diagram with error bars (n=3).

**S18 Lysosome Staining Experiment**

HeLa cells at an appropriate density were seeded into 60 mm-diameter culture dishes, followed by the addition of 2 mL of DMEM medium. Subsequently, different nanoparticles were introduced into the medium, with the final concentrations of gold nanostars (AuNS) and nanodiamonds (ND) adjusted to 1.8 pM and 6×10⁻⁴ v%, respectively. The dishes were gently shaken to ensure homogeneous dispersion of the nanoparticles in the DMEM medium. The culture dishes were then incubated overnight in a CO₂ incubator to facilitate sufficient cellular internalization of the nanoparticles.On the following day, 5 μL of Lyso-Tracker Green (a lysosomal dye) was added to the medium, and the dishes were shaken thoroughly before being placed back into the CO₂ incubator for a 30-min incubation. After incubation, the cells were rinsed three times with phosphate-buffered saline (PBS) to remove excess extracellular dye. Finally, the cells were observed under a microscope: lysosomes were visualized using the fluorescence mode, ND were observed via the total internal reflection fluorescence (TIRF) mode, and AuNS were examined under the dark-field mode.


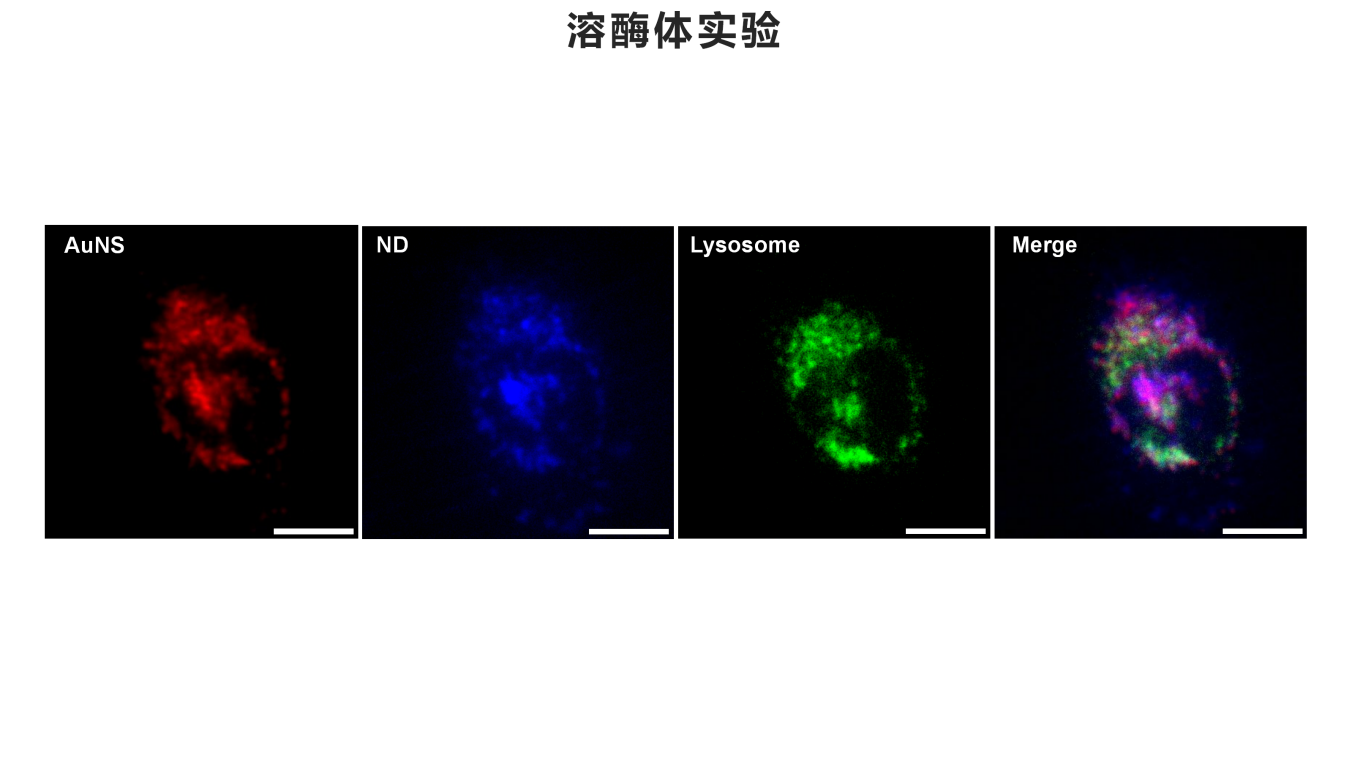


***Figure S21.*** Three-color co-localization images of AuNS, ND and lysosomes. The red signal corresponds to AuNS, the blue signal to ND, and the green signal to lysosomes. Scale bars: 20 μm.

**S19 Cell Photothermal Experiment**

Hela cells at an appropriate density were seeded in a Petri dish (Diameter 35 mm), and then 1 ml of DMEM medium was added. Subsequently, different nanoparticles (with the final concentration of AuNS being 1.8 pM and that of ND being 7.5×10⁻⁴ v%) were added into the media of different groups. The mixtures were shaken evenly to ensure that the nanoparticles were uniformly distributed in the DMEM medium. Then, they were placed in a carbon dioxide incubator and cultured overnight so that the nanoparticles could be fully endocytosed by the cells.

On the second day, the middle position of the medium was irradiated with a near-infrared laser at 808 nm (at a height of 15 cm) for 6 minutes, with the irradiated area being a circular region with a diameter of 25 mm. During laser irradiation, two thermocouples were immersed in the culture medium without touching the bottom of the culture dish for real-time monitoring of the medium temperature. After that, the samples were put back into the carbon dioxide incubator and continued to be cultured for another 4 hours. After the culturing was completed, the Hela cells were fluorescently stained with Calcein-AM. Once the staining was finished, the survival status of the cells in the irradiated area was observed under a microscope. The fluorescence signals of the cells in different fields of view were detected using Image J software. Then, the cell survival rate was calculated, statistically analyzed, and a difference analysis was carried out.


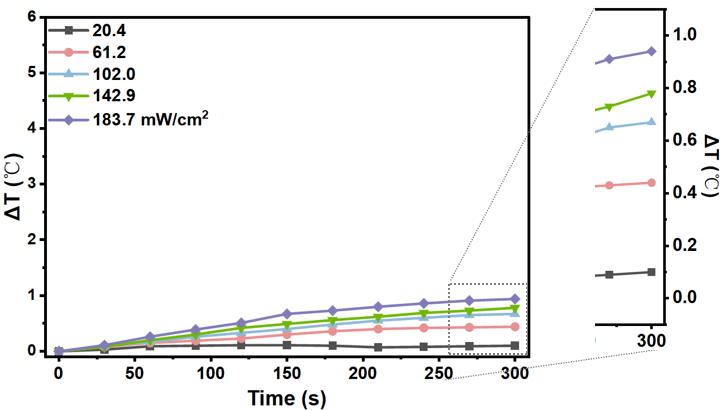


***Figure S22.*** Temperature Change Curves of the Medium in AuNS/ND Group under Irradiation with Different Power Densities.

**S20 Calcein Staining of Cell**

Calcein-AM (final concentration: 1 nM) was added to the culture medium containing adherent cells. The mixture was gently agitated to ensure homogeneity and then incubated at a constant temperature in a CO₂ incubator for 30 minutes to facilitate the efficient uptake of calcein into the cells. Following incubation, the calcein-containing medium was aspirated, and the cells were thoroughly washed three times with phosphate-buffered saline (PBS) to remove excess dye. Phenol red-free culture medium was subsequently added to the dish to avoid interference with subsequent fluorescence imaging. The cells were then returned to the CO₂ incubator for an additional 20-minute incubation period. Finally, cellular imaging was performed under a microscope.

**S21 Animal Experiments**

**S21.1 Animals and Tumor Models**

Adult female BALB/c nude mice (6-8 weeks old) were purchased from the Animal Center of Wuhan University. All animal experiments were conducted in accordance with the guidelines of the Institutional Animal Care and Use Committee and approved by the Animal Ethics Committee of Wuhan University. Mice were housed under controlled conditions (20-25°C, 12 h light/dark cycle) with free access to food and water. To establish subcutaneous xenograft tumor models, human B-cell lymphoma ramos cells (Cell Bank of the Chinese Academy of Sciences) in the logarithmic growth phase were harvested, trypsinized, and resuspended in ice-cold PBS. The cell suspension was mixed with Matrigel (40186ES08, YEASON, Shanghai, China) at a 1:1 volume ratio on ice to prevent gelation. A total volume of 200 µL containing 1×10⁷ cells was subcutaneously injected into the right armpit of each mouse. Tumor growth was monitored daily using a digital caliper, and tumor volume (V) was calculated using the formula: V = 0.5 × length × width². When the tumors reached an average volume of approximately 50-100 mm³ (about 7-10 days post-inoculation), the mice were randomly divided into experimental groups (n = 5 per group). The groups included PBS group, ND group, AuNS group, and AuNS+ND group, which received intratumoral injections of PBS (50 µL/100 mm³), ND (10 mg/mL, 10 µL/100 mm³), AuNS (200 pM, 50 µL/100 mm³), and AuNS+ND mixture, respectively. Twenty-four hours post-injection, tumors were irradiated with an 808 nm laser (500 mW/cm²) for 6 minutes. Photothermal therapy was performed daily for 5 consecutive days. Body weight and tumor volume were monitored daily. Tumor tissues (n = 5 per group) were excised 5 days after the first treatment and fixed in 4% paraformaldehyde.

**S21.2 In Vivo Photothermal Therapy**

Mice were anesthetized with 2% isoflurane and placed on a warming pad. The tumor regions were shaved for clear laser exposure. Mice in each group received a single intratumoral injection of PBS (50 µL/100 mm³), ND (10 mg/mL, 10 µL/100 mm³), AuNS (200 pM, 50 µL/100 mm³), or AuNS+ND mixture. Twenty-four hours post-injection, tumors were irradiated with an 808 nm NIR laser at a power density of 500 mW/cm² for 6 minutes. This irradiation procedure was repeated once daily for 5 consecutive days. During the first irradiation session, the temperature change inside the tumor was monitored in real time using a temperature probe.

**S21.3 Paraffin Embedding, Sectioning, and H&E Staining**

Tumor tissues were fixed in 4% paraformaldehyde for 24 h, dehydrated through graded ethanol series, cleared in xylene, and embedded in paraffin. Sections (4-5 µm thick) were cut using a microtome and mounted on glass slides. For H&E staining, sections were deparaffinized, rehydrated, stained with hematoxylin and eosin following standard protocols, dehydrated, cleared, and mounted. Histopathological evaluation was performed under a light microscope.

**S21.4 Frozen Section Preparation**

For frozen sections, fresh tumor tissues were embedded in optimal cutting temperature (OCT) compound, snap-frozen in liquid nitrogen, and stored at -80°C. Sections (8-10 µm thick) were cut using a cryostat at -20°C, mounted on pre-cooled slides, and stored at -80°C until use.


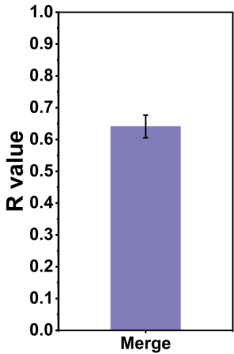


***Figure S23.*** Co-localization coefficient analysis of AuNS and ND in tumor cryosections. n=4.


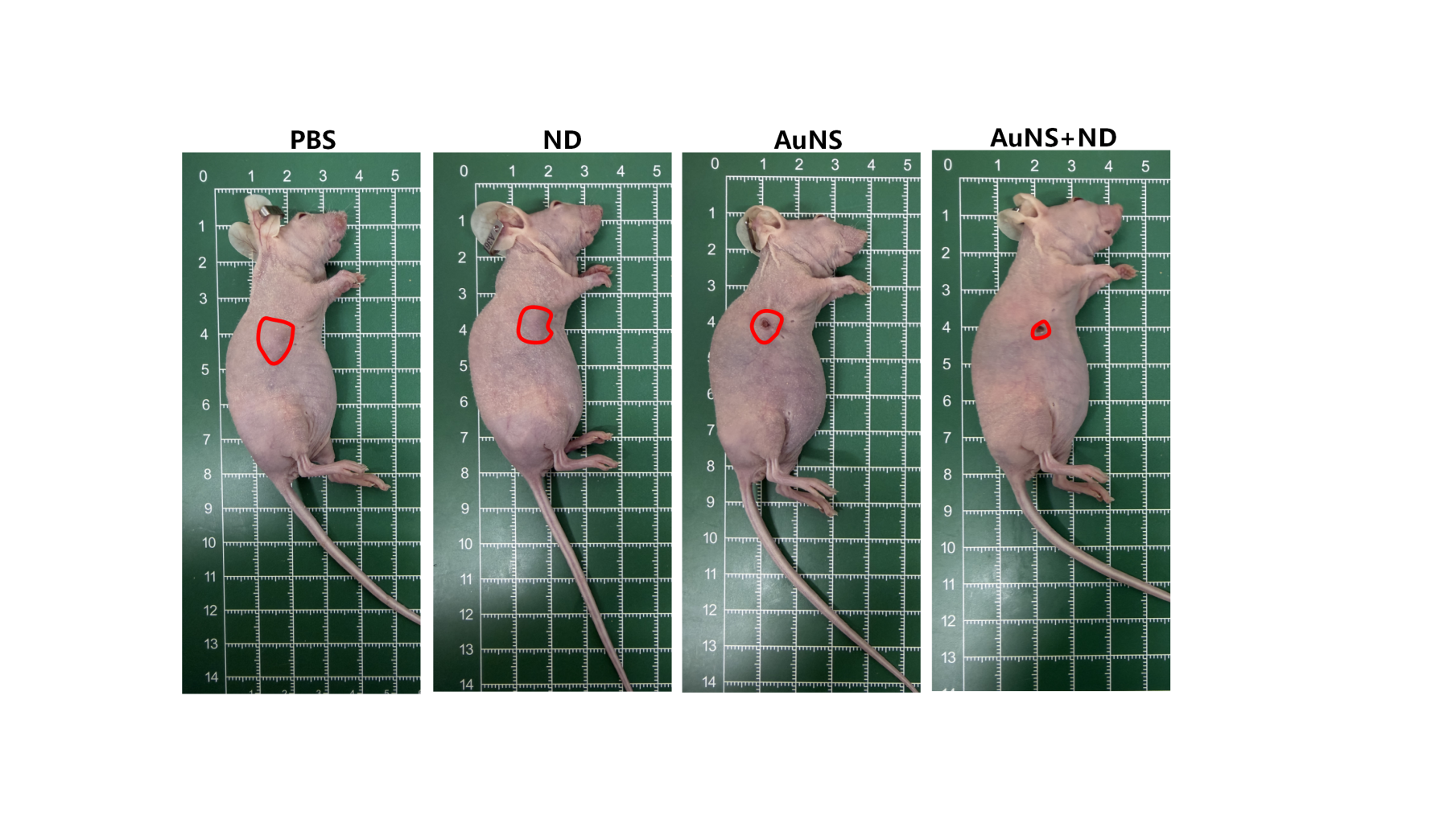


***Figure S24.*** Representative photographs of mice after treatment. Representative photographs of tumor-bearing mice from the PBS, ND, AuNS, and AuNS+ND groups after the in vivo photothermal therapy. The tumor region is outlined in red. Mice received intratumoral injection of the indicated formulations and were irradiated with an 808 nm laser (500 mW/cm², 6 min) once per day for 5 consecutive days.


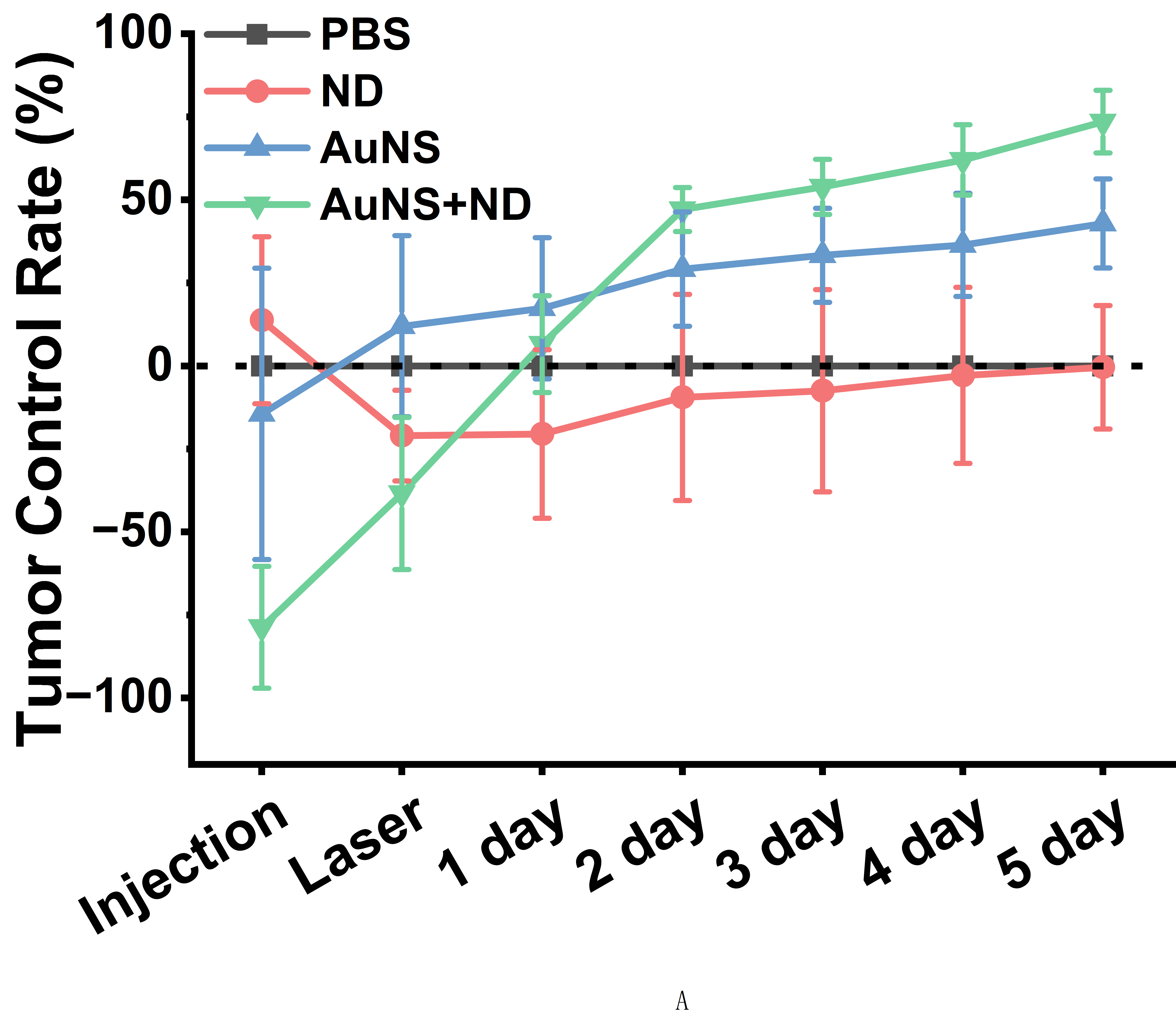


***Figure S25.*** Tumor control rate (TCR) of different treatment groups during the 5-day therapy regimen. TCR was calculated as [(V_PBS_ − V_treat_)/V_PBS_ × 100%], where V_PBS_ is the mean tumor volume of the PBS group and V_treat_ is the mean tumor volume of the corresponding treatment group at the same time point. Data are mean ± SD (n = 5).


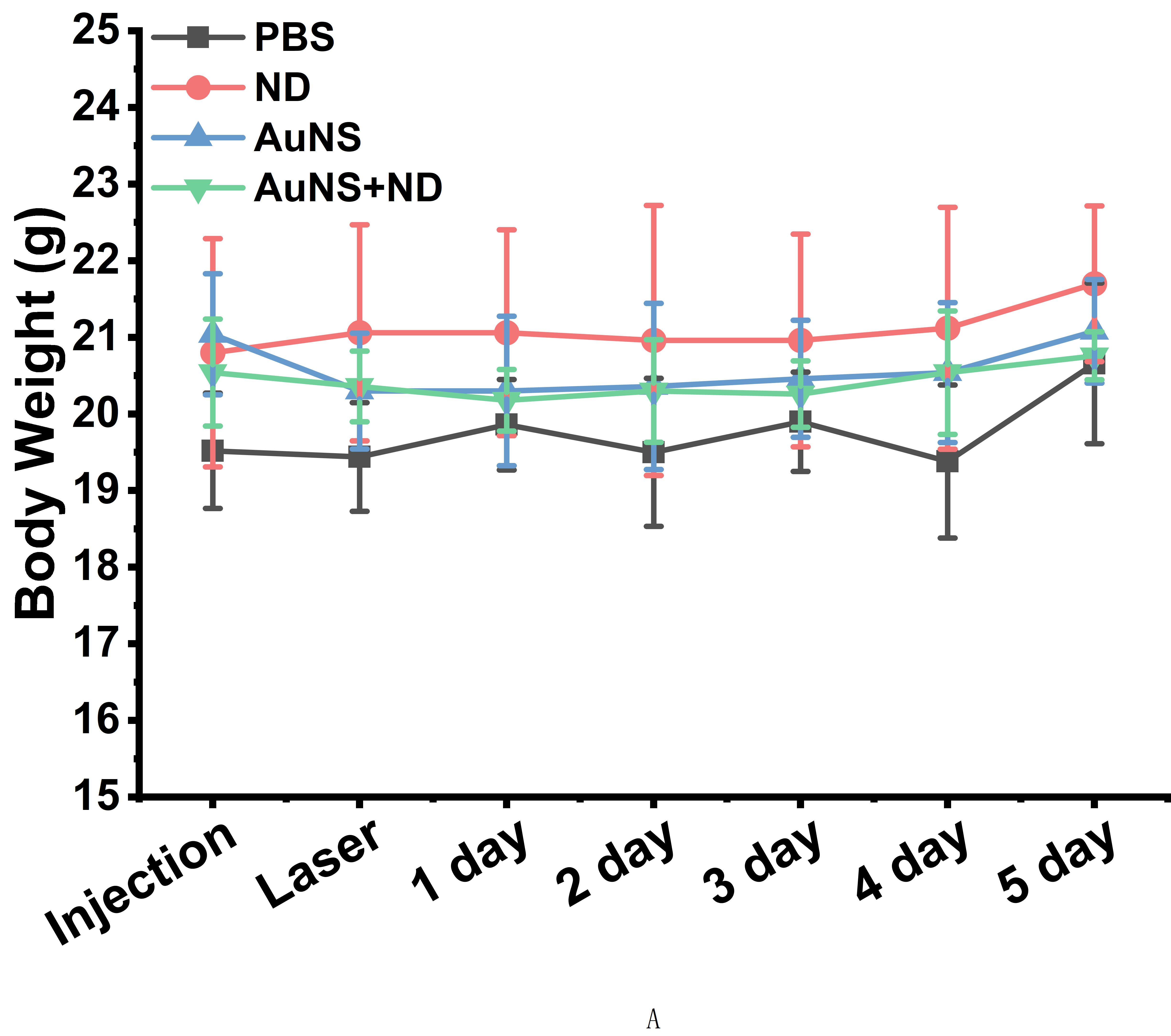


***Figure S26.*** Body weight changes of tumor-bearing mice in the PBS, ND, AuNS, and AuNS+ND groups during the in vivo treatment period. Data are mean ± SD (n = 5), indicating no obvious systemic toxicity under the applied treatment conditions.

***
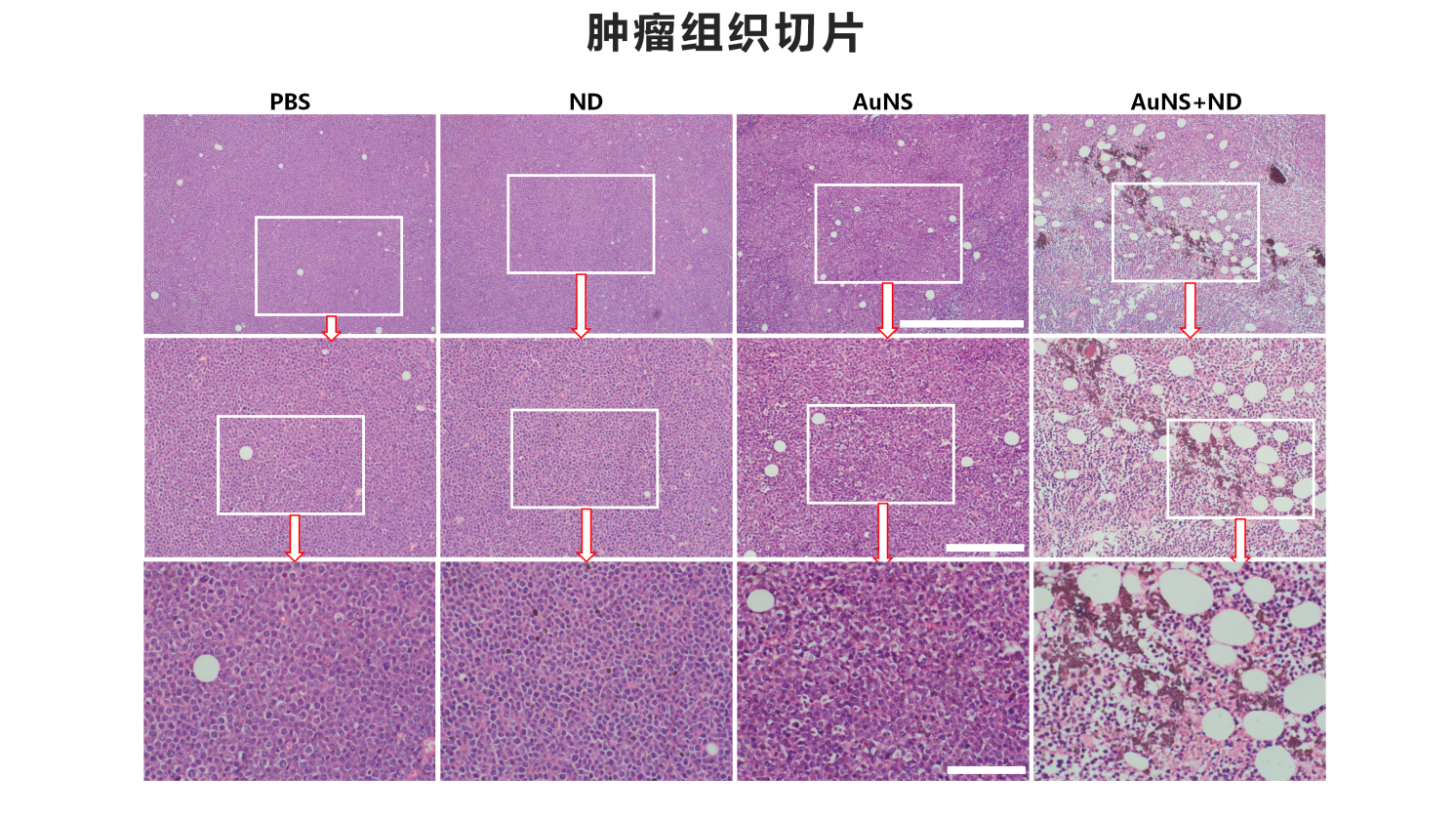
***

***Figure S27.*** Representative H&E-stained tumor sections collected after the in vivo therapy from the PBS, ND, AuNS, and AuNS+ND groups.The second and third rows are magnified views of the boxed regions in the first and second rows, respectively. Scale bars: 400 μm/200 μm/100 μm.

**Statistical Analysis**

All quantitative data are presented as mean ± standard deviation (SD), unless otherwise stated. No data points were excluded from the analysis unless explicitly indicated in the corresponding figure legend. For microscopy image analysis, raw images were processed using identical parameters across experimental and control groups. Regions of interest (ROIs) were defined using the same criteria for each dataset, and background signals were measured from cell-/sample-free areas and subtracted when applicable. Image-based quantification (e.g., fluorescence intensity, colocalization analysis, and/or area measurement) was performed using Fiji (Windows x64). Plotting and statistical calculations were performed using Origin.

Sample size (n) is reported in each figure legend and refers to the number of independent biological replicates unless otherwise specified; for image-based measurements, multiple fields of view were analyzed per replicate, and the averaged value per replicate was used for statistics. Comparisons between two groups were evaluated using an unpaired two-tailed Student’s t-test. Comparisons among three or more groups were evaluated using one-way analysis of variance (ANOVA) followed by an appropriate post-hoc multiple-comparison test. A P value < 0.05 was considered statistically significant. Significance levels are indicated as P < 0.05 (*), P < 0.01 (**), P < 0.001 (***).

**References**

1. Jacques SL. Optical properties of biological tissues: a review. *Phys Med Biol*. 2013;58(11):R37–R61. DOI:10.1088/0031-9155/58/11/R37.
2. Hale GM, Querry MR. Optical constants of water in the 200 nm to 200 µm wavelength region. *Appl Opt*. 1973;12(3):555–563. DOI:10.1364/AO.12.000555.
3. Li Y, Ma J, Ma Z. Synthesis of gold nanostars with tunable morphology and their electrochemical application for hydrogen peroxide sensing. *Electrochimica Acta.* 2013, 108, 435-440. DOI: [10.1016/j.electacta.2013.06.141](https://www.x-mol.com/paperRedirect/4226488).
